# Supplementary material for: SARs for the Antiparasitic Plant Metabolite Pulchrol. 3. Combinations of New Substituents in A/B-Rings and A/C-Rings
Source: Molecules. 2021 Jun 28;26(13):3944. doi: 10.3390/molecules26133944 (PMC8271509; doi:10.3390/molecules26133944)
Supplement: Supplementary file 1 [file molecules-26-03944-s001.zip › molecules-1174607-supplementary.pdf]

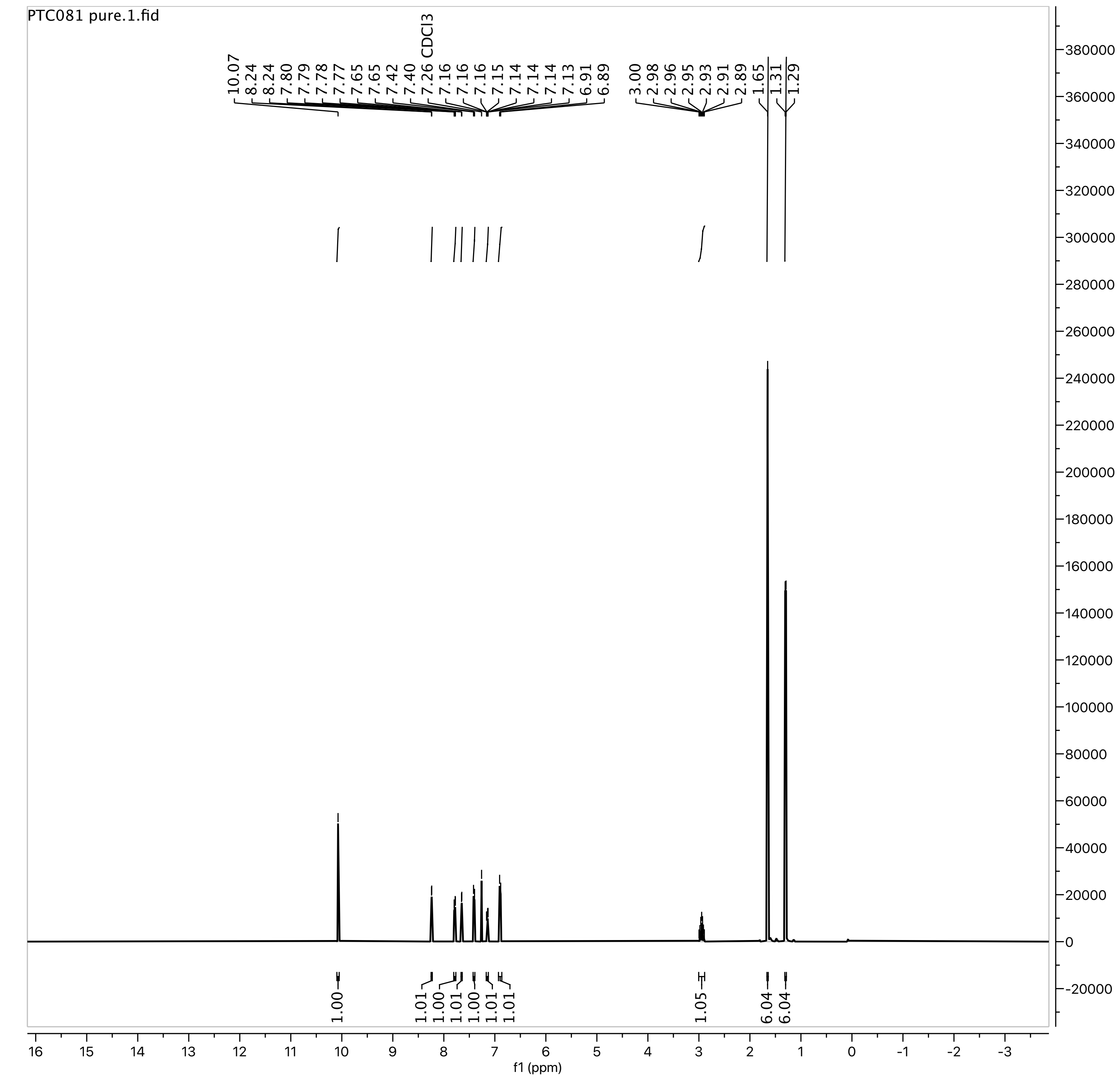

	Parameter	Value
1	Solvent	CDCl3
2	Experiment	1D
3	Spectrometer Frequency	400.13
4	Nucleus	1H

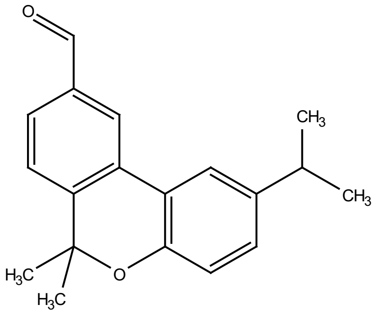

^{1}H NMR (400 MHz, $CDCl_3$) δ 10.07 (s, 1H), 8.24 (d, *J* = 1.7 Hz, 1H), 7.79 (dd, *J* = 7.9, 1.6 Hz, 1H), 7.65 (d, *J* = 2.2 Hz, 1H), 7.41 (d, *J* = 7.9 Hz, 1H), 7.15 (ddd, *J* = 8.3, 2.2, 0.5 Hz, 1H), 6.90 (d, *J* = 8.3 Hz, 1H), 2.95 (hept, *J* = 7.0 Hz, 1H), 1.65 (s, 6H), 1.30 (d, *J* = 6.9 Hz, 6H).

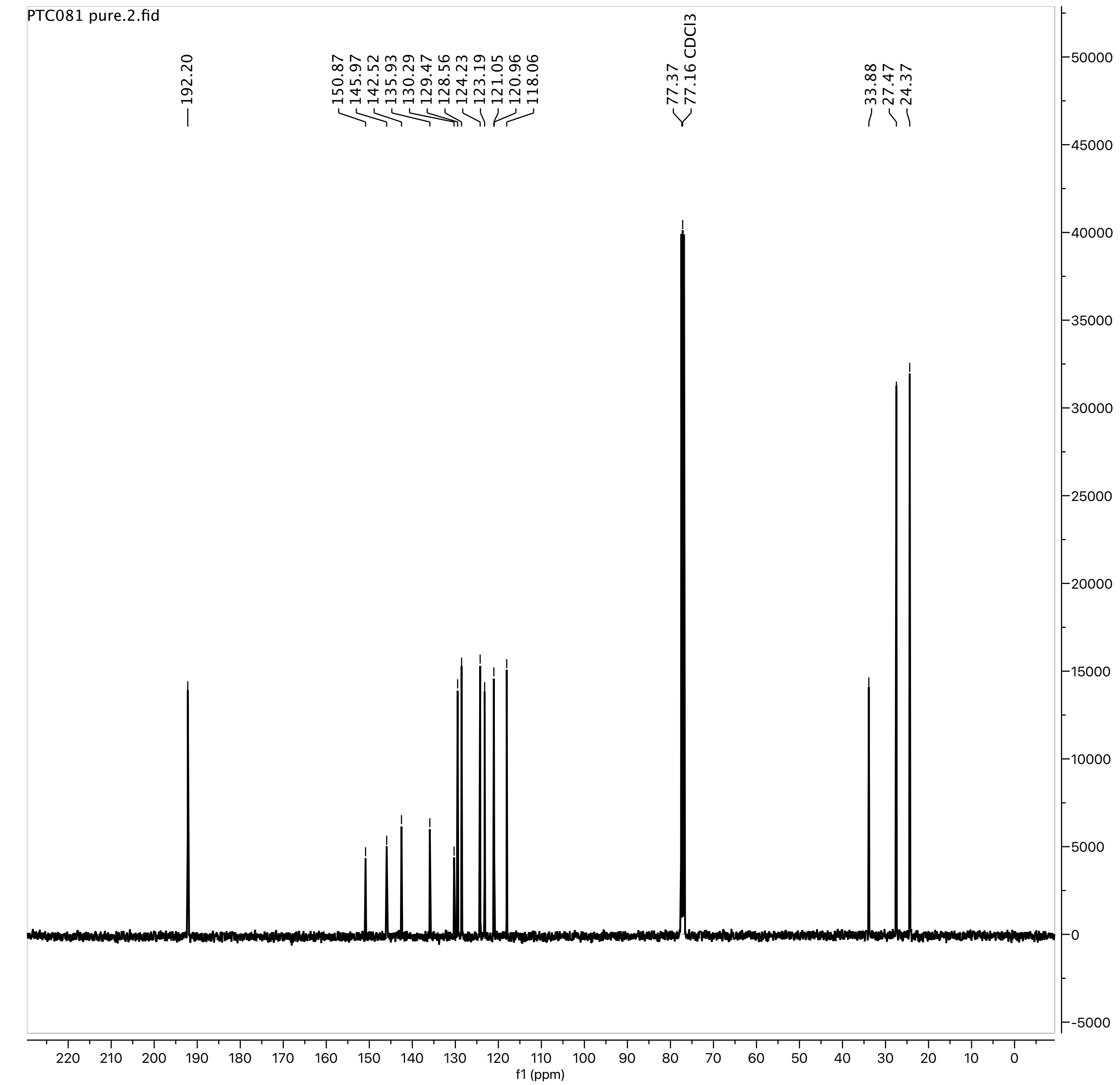

	Parameter	Value
1	Solvent	CDCl3
2	Experiment	1D
3	Spectrometer Frequency	100.62
4	Nucleus	13C

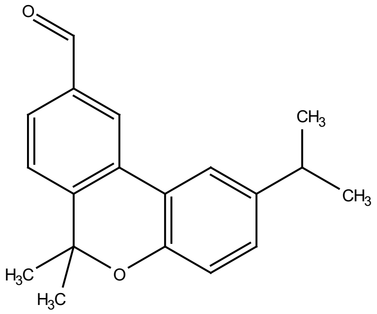

^{13}C NMR (101 MHz, $CDCl_3$) δ 192.20, 150.87, 145.97, 142.52, 135.93, 130.29, 129.47, 128.56, 124.23, 123.19, 121.05, 120.96, 118.06, 77.37, 33.88, 27.47, 24.37.

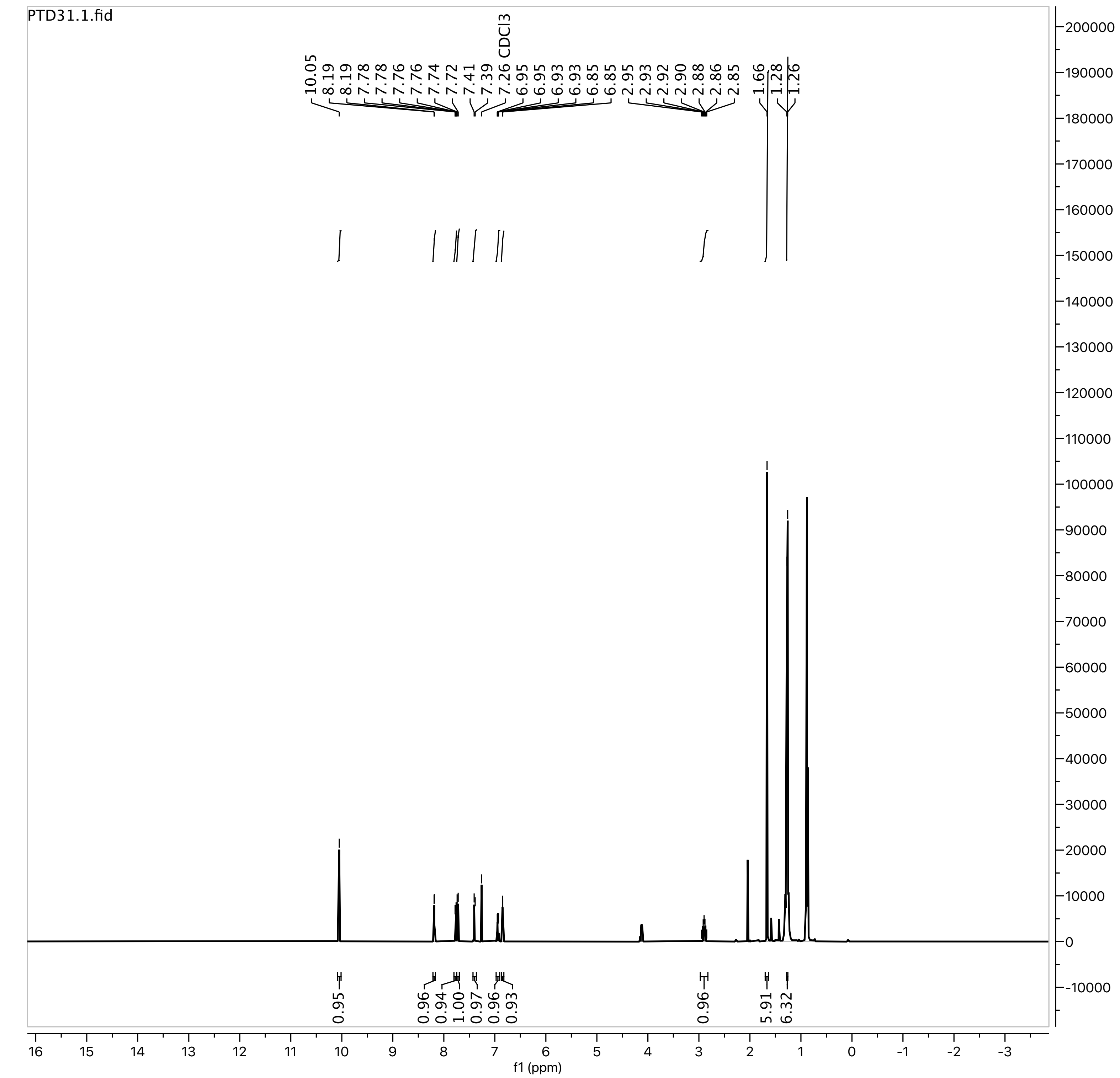

	Parameter	Value
1	Solvent	CDCl3
2	Experiment	1D
3	Spectrometer Frequency	400.13
4	Nucleus	1H

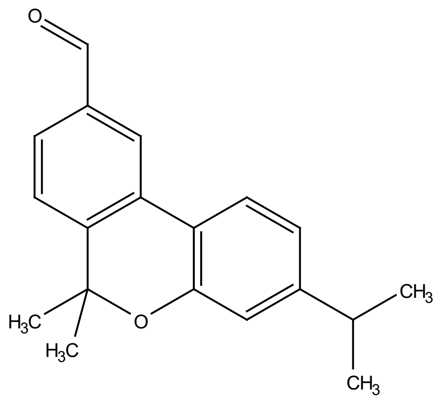

^{1}H NMR (400 MHz, $CDCl_3$) δ 10.05 (s, 1H), 8.19 (d, J = 1.6 Hz, 1H), 7.77 (dd, J = 7.9, 1.6 Hz, 1H), 7.73 (d, J = 8.0 Hz, 1H), 7.40 (d, J = 7.9 Hz, 1H), 6.94 (dd, J = 8.0, 1.8 Hz, 1H), 6.85 (d, J = 1.7 Hz, 1H), 2.90 (hept, J = 7.0 Hz, 1H), 1.66 (s, 6H), 1.27 (d, J = 6.9 Hz, 6H).

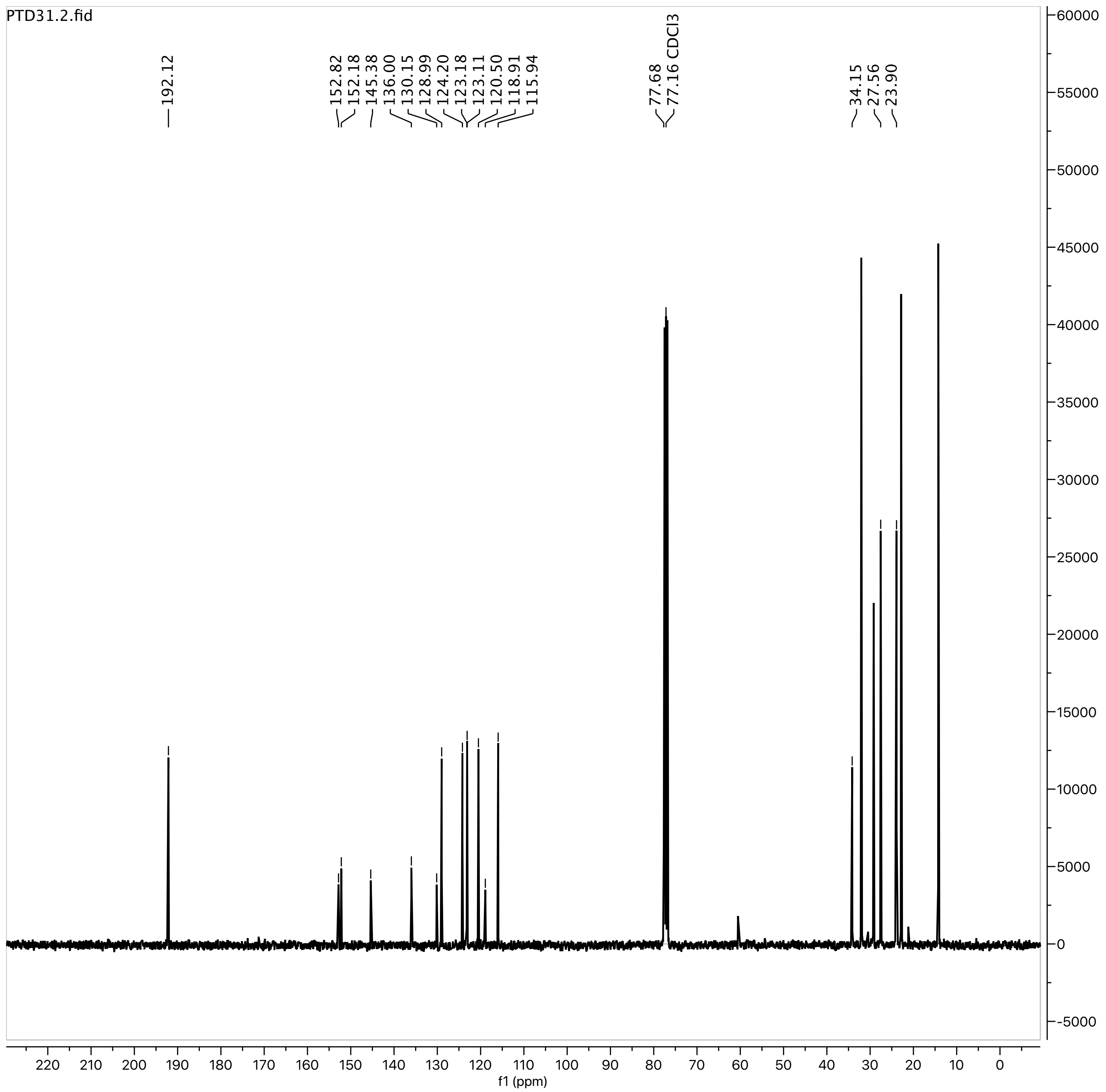

	Parameter	Value
1	Solvent	CDCl3
2	Experiment	1D
3	Spectrometer Frequency	100.62
4	Nucleus	13C

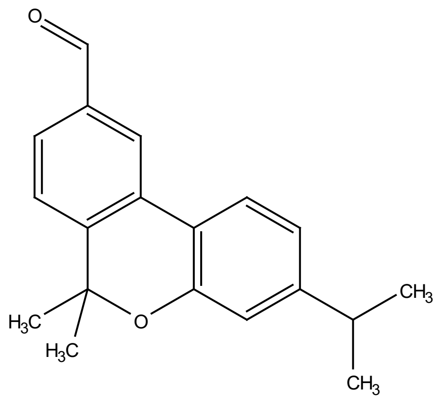

^{13}C NMR (101 MHz, $CDCl_3$) δ 192.12, 152.82, 152.18, 145.38, 136.00, 130.15, 128.99, 124.20, 123.18, 123.11, 120.50, 118.91, 115.94, 77.68, 34.15, 27.56, 23.90.

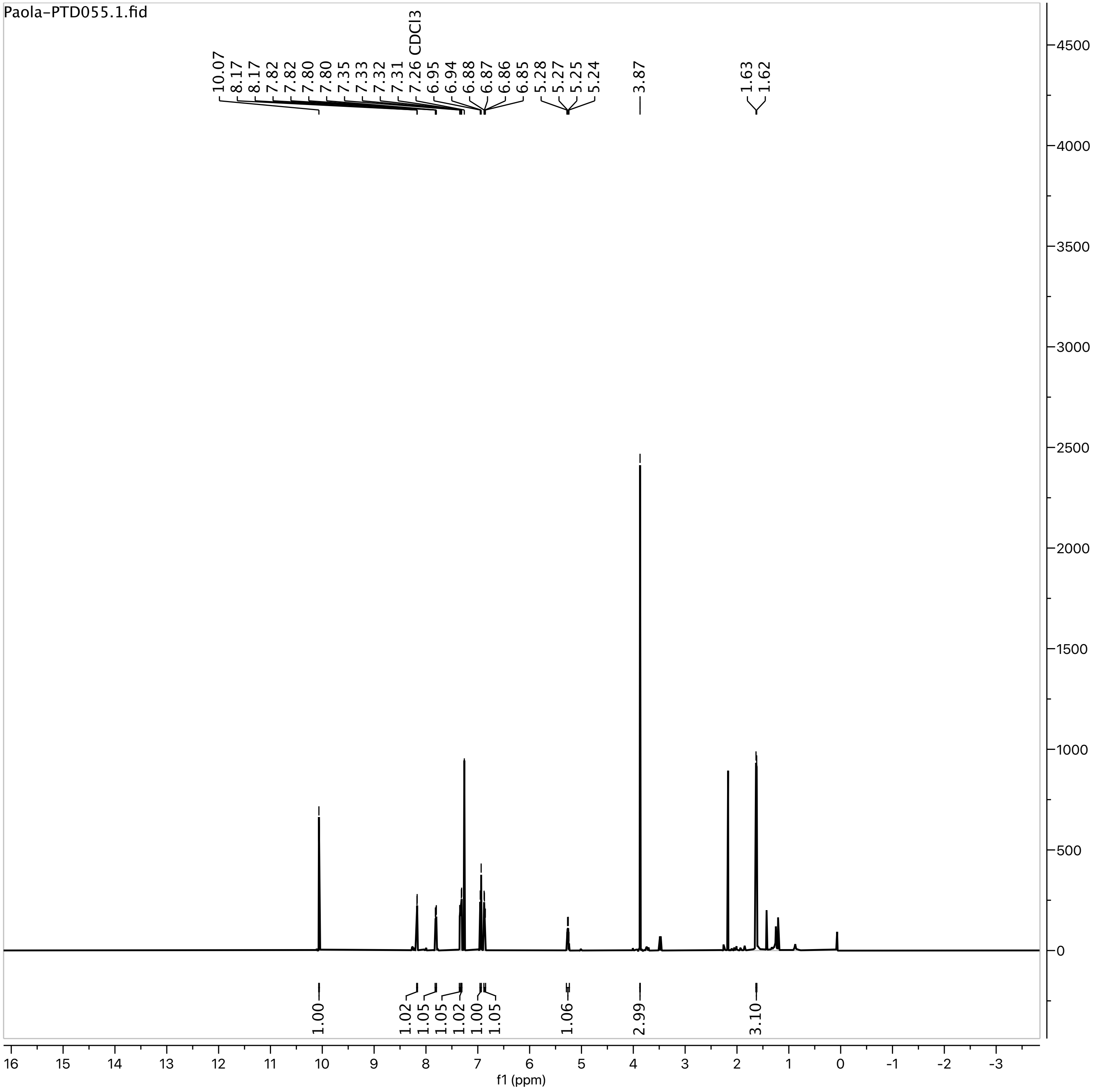

	Parameter	Value
1	Solvent	CDCl3
2	Experiment	1D
3	Spectrometer Frequency	500.17
4	Nucleus	1H

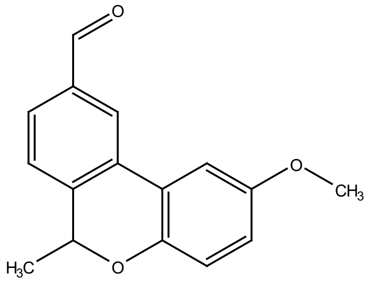

^{1}H NMR (500 MHz, $CDCl_3$) δ 10.07 (s, 1H), 8.17 (d, *J* = 1.5 Hz, 1H), 7.81 (dd, *J* = 7.7, 1.6 Hz, 1H), 7.34 (d, *J* = 7.8 Hz, 1H), 7.31 (d, *J* = 2.9 Hz, 1H), 6.94 (d, *J* = 8.7 Hz, 1H), 6.87 (dd, *J* = 8.9, 2.9 Hz, 1H), 5.29 – 5.23 (m, 1H), 3.87 (s, 3H), 1.63 (d, *J* = 6.7 Hz, 3H).

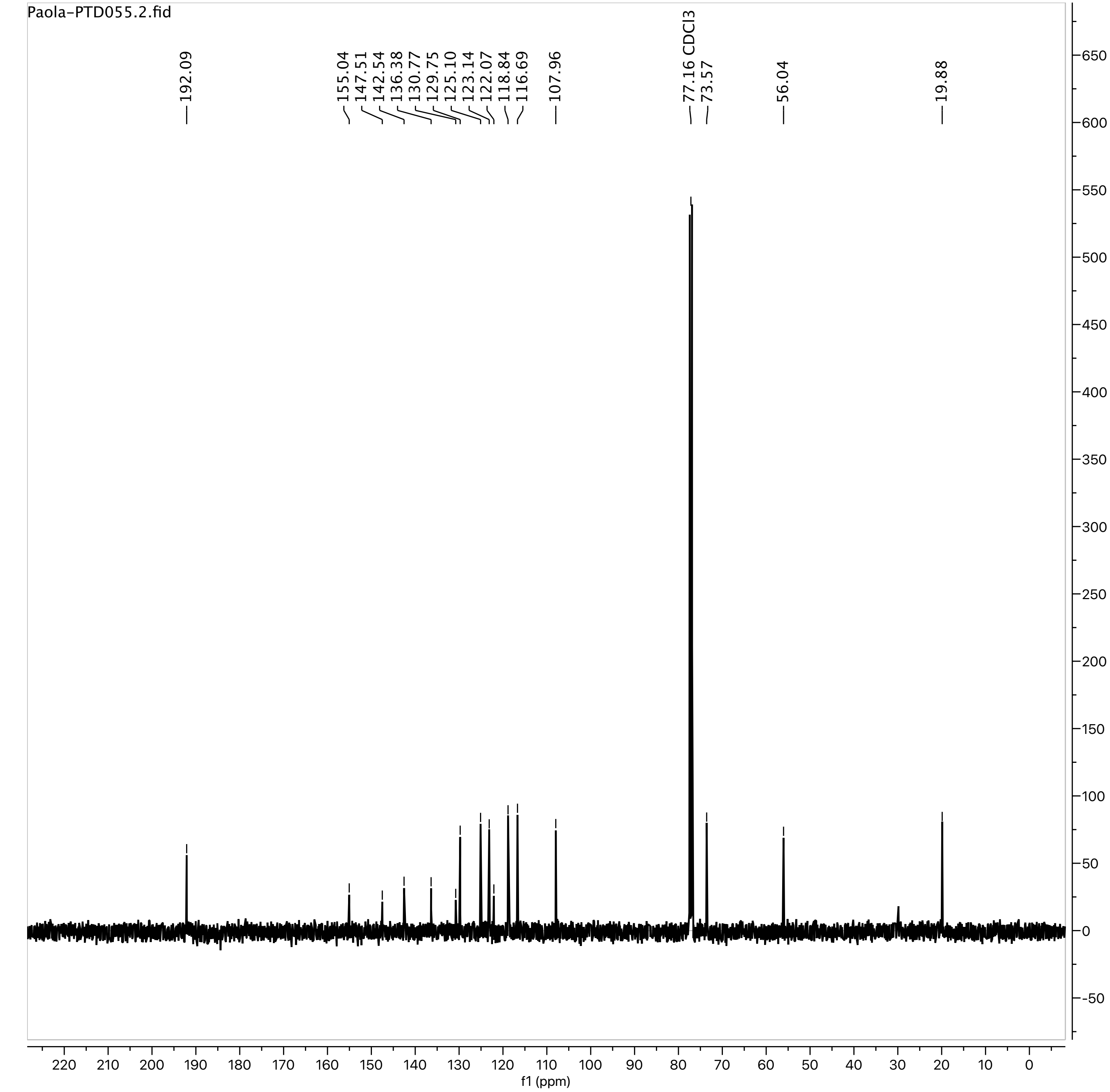

	Parameter	Value
1	Solvent	CDCl3
2	Experiment	1D
3	Spectrometer Frequency	125.78
4	Nucleus	13C

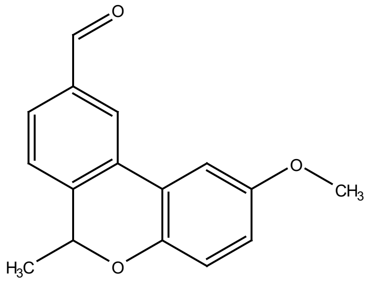

^{13}C NMR (126 MHz, $CDCl_3$) δ 192.09, 155.04, 147.51, 142.54, 136.38, 130.77, 129.75, 125.10, 123.14, 122.07, 118.84, 116.69, 107.96, 73.57, 56.04, 19.88.

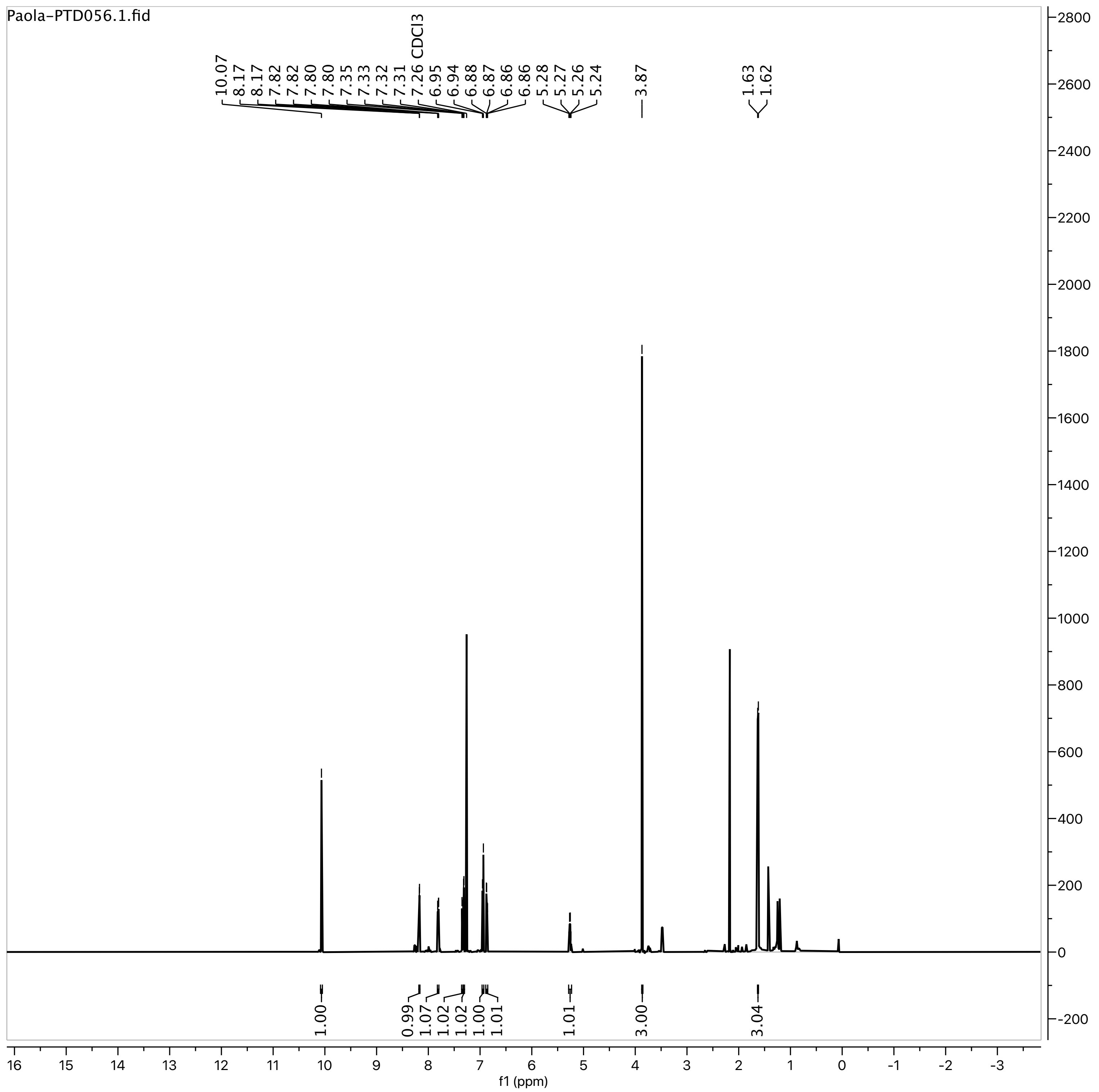

	Parameter	Value
1	Solvent	CDCl3
2	Experiment	1D
3	Spectrometer Frequency	500.17
4	Nucleus	1H

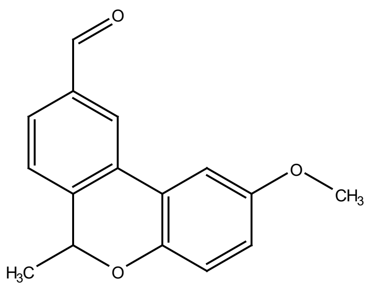

^{1}H NMR (500 MHz, $CDCl_3$) δ 10.07 (s, 1H), 8.17 (d, J = 1.7 Hz, 1H), 7.81 (dd, J = 7.8, 1.5 Hz, 1H), 7.34 (d, J = 7.6 Hz, 1H), 7.32 (d, J = 3.1 Hz, 1H), 6.94 (d, J = 8.9 Hz, 1H), 6.87 (dd, J = 8.8, 3.0 Hz, 1H), 5.29 – 5.23 (m, 1H), 3.87 (s, 3H), 1.63 (d, J = 6.6 Hz, 3H).

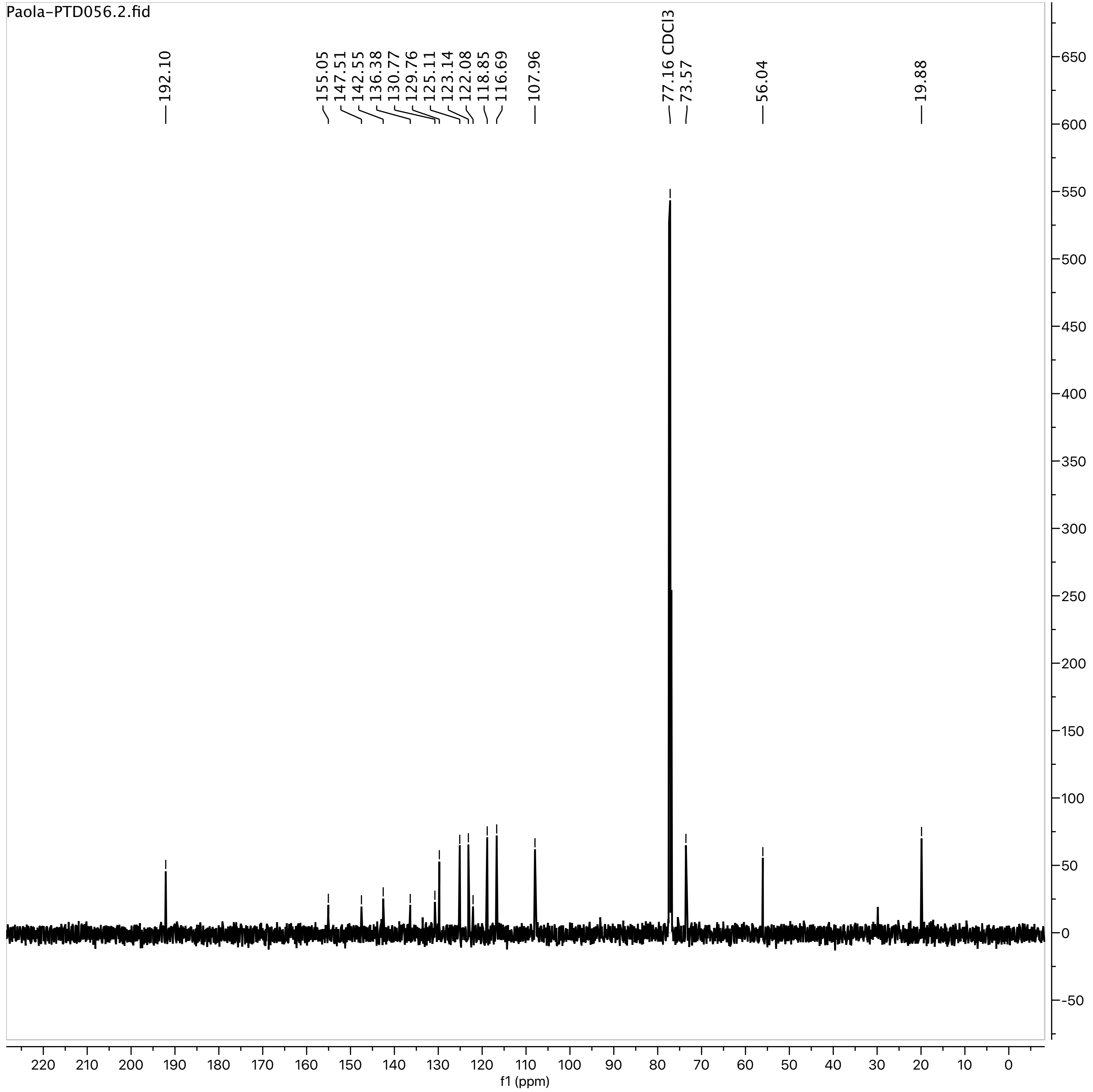

	Parameter	Value
1	Solvent	CDCl3
2	Experiment	1D
3	Spectrometer Frequency	125.78
4	Nucleus	13C

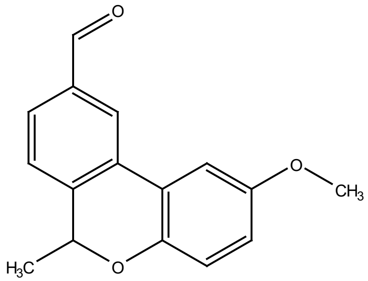

^{13}C NMR (126 MHz, $CDCl_3$) δ 192.10, 155.05, 147.51, 142.55, 136.38, 130.77, 129.76, 125.11, 123.14, 122.08, 118.85, 116.69, 107.96, 73.57, 56.04, 19.88.

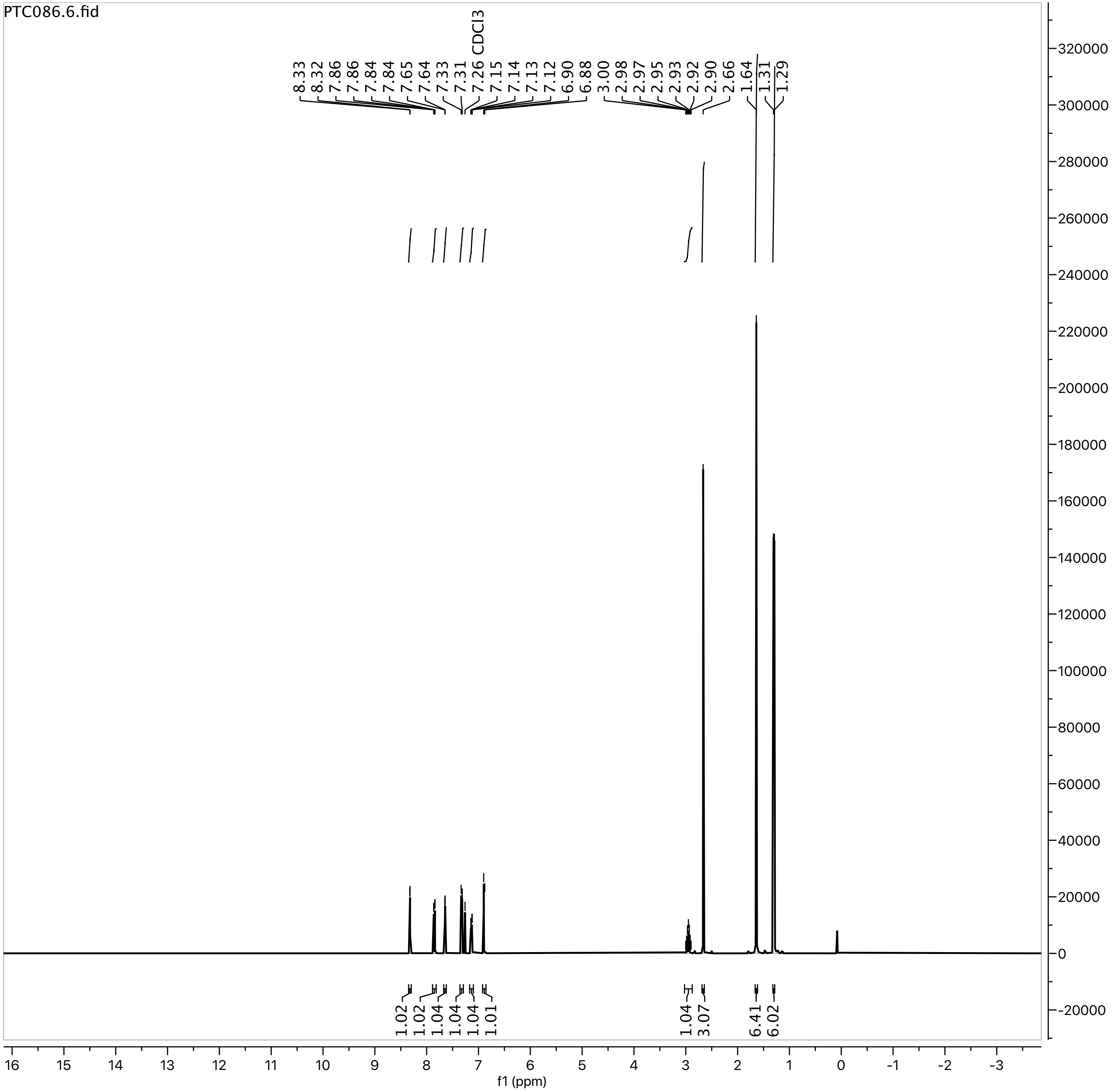

	Parameter	Value
1	Solvent	CDCl3
2	Experiment	1D
3	Spectrometer Frequency	400.13
4	Nucleus	1H

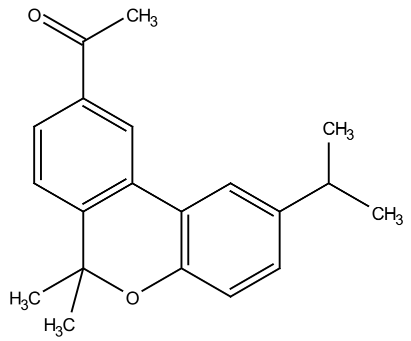

^{1}H NMR (400 MHz, $CDCl_3$) δ 8.32 (d, J = 1.8 Hz, 1H), 7.85 (dd, J = 8.1, 1.8 Hz, 1H), 7.65 (d, J = 2.2 Hz, 1H), 7.32 (d, J = 8.1 Hz, 1H), 7.13 (dd, J = 8.3, 2.1 Hz, 1H), 6.89 (d, J = 8.3 Hz, 1H), 2.95 (hept, J = 6.9 Hz, 1H), 2.66 (s, 3H), 1.64 (s, 6H), 1.30 (d, J = 7.0 Hz, 6H).

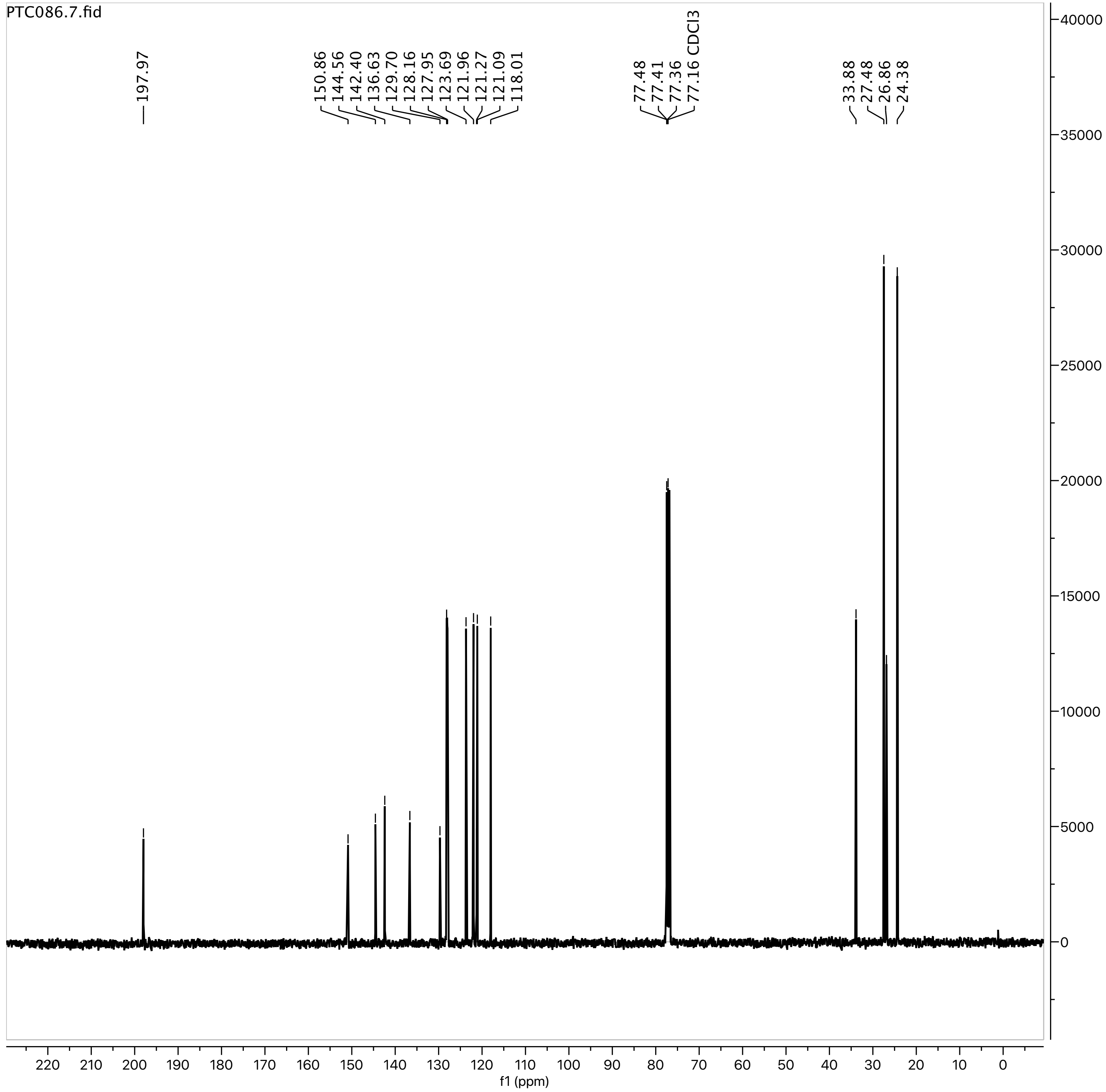

	Parameter	Value
1	Solvent	CDCl3
2	Experiment	1D
3	Spectrometer Frequency	100.62
4	Nucleus	13C

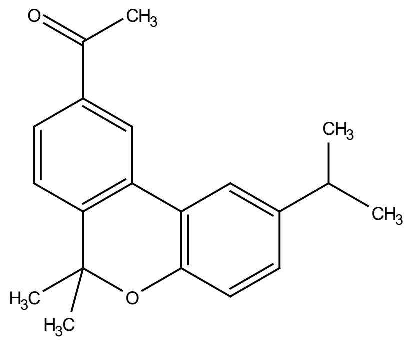

^{13}C NMR (101 MHz, $CDCl_3$) δ 197.97, 150.86, 144.56, 142.40, 136.63, 129.70, 128.16, 127.95, 123.69, 121.96, 121.27, 121.09, 118.01, 77.44 (d, J = 7.4 Hz), 33.88, 27.48, 26.86, 24.38.

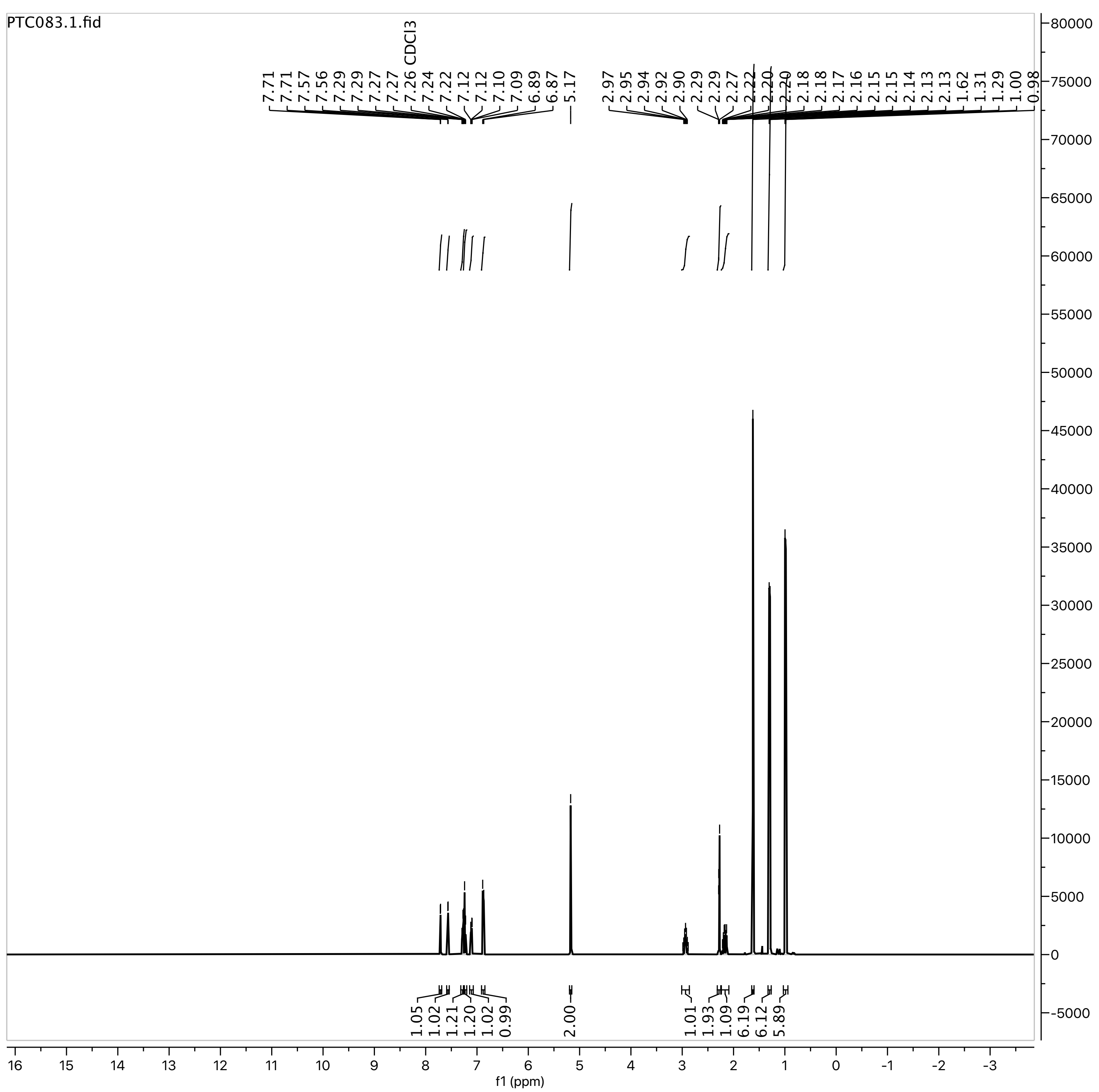

	Parameter	Value
1	Solvent	CDCl3
2	Experiment	1D
3	Spectrometer Frequency	400.13
4	Nucleus	1H

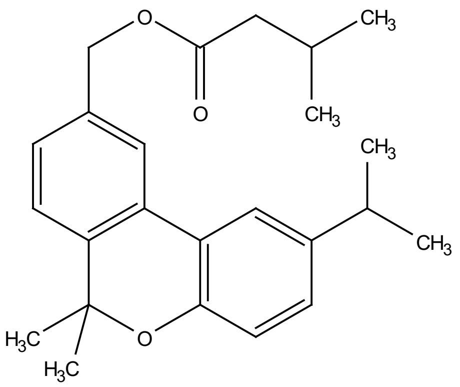

^{1}H NMR (400 MHz, $CDCl_3$) δ 7.71 (d, *J* = 1.7 Hz, 1H), 7.57 (d, *J* = 2.2 Hz, 1H), 7.28 (dd, *J* = 7.9, 1.7 Hz, 1H), 7.23 (d, *J* = 8.0 Hz, 1H), 7.11 (dd, *J* = 8.3, 2.1 Hz, 1H), 6.88 (d, *J* = 8.3 Hz, 1H), 5.17 (s, 2H), 2.94 (hept, *J* = 7.0 Hz, 1H), 2.32 – 2.25 (m, 2H), 2.24 – 2.09 (m, 1H), 1.62 (s, 6H), 1.30 (d, *J* = 6.9 Hz, 6H), 0.99 (d, *J* = 6.6 Hz, 6H).

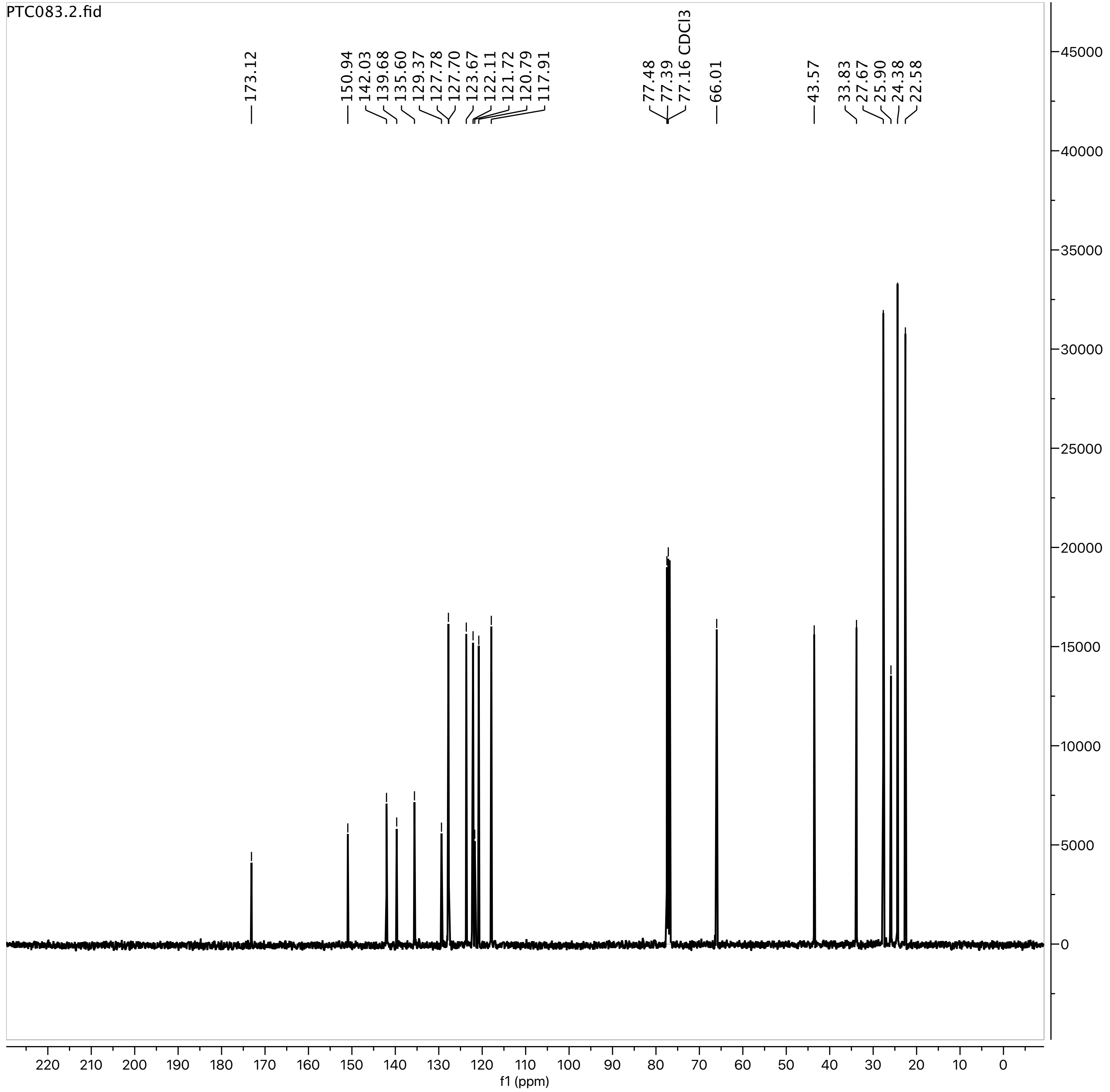

	Parameter	Value
1	Solvent	CDCl3
2	Experiment	1D
3	Spectrometer Frequency	100.62
4	Nucleus	13C

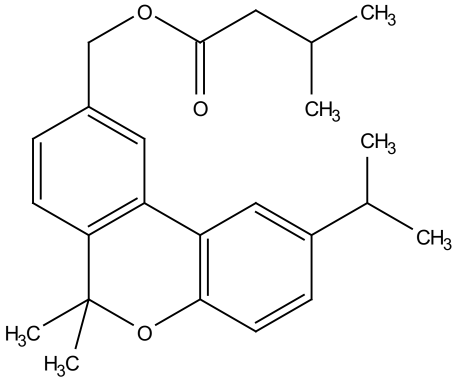

^{13}C NMR (101 MHz, $CDCl_3$) δ 173.12, 150.94, 142.03, 139.68, 135.60, 129.37, 127.78, 127.70, 123.67, 122.11, 121.72, 120.79, 117.91, 77.43 (d, J = 9.1 Hz), 66.01, 43.57, 33.83, 27.67, 25.90, 24.38, 22.58.

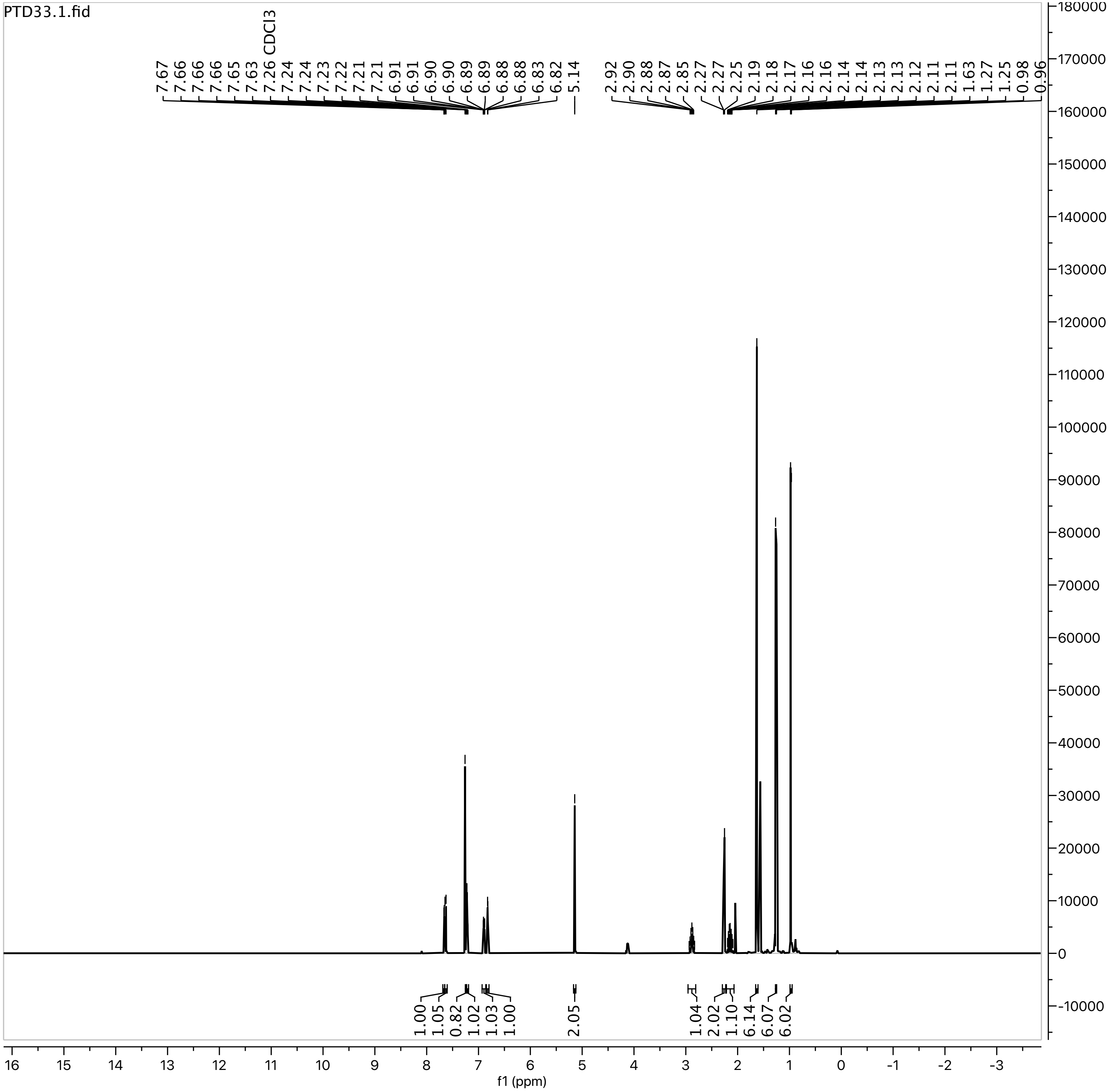

	Parameter	Value
1	Solvent	CDCl3
2	Experiment	1D
3	Spectrometer Frequency	400.13
4	Nucleus	1H

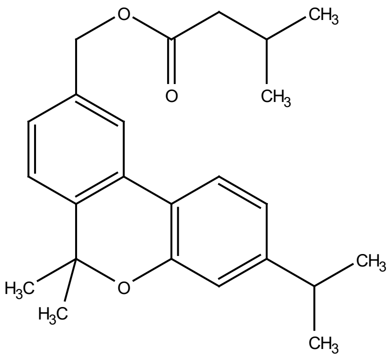

^{1}H NMR (400 MHz, $CDCl_3$) δ 7.69 – 7.65 (m, 1H), 7.64 (d, J = 8.0 Hz, 1H), 7.24 (d, J = 1.7 Hz, 1H), 7.22 (dd, J = 7.9, 0.6 Hz, 1H), 6.90 (ddd, J = 8.0, 1.8, 0.5 Hz, 1H), 6.82 (d, J = 2.0 Hz, 1H), 5.14 (s, 2H), 2.96 – 2.81 (m, 1H), 2.29 – 2.23 (m, 2H), 2.22 – 2.07 (m, 1H), 1.63 (s, 6H), 1.26 (d, J = 6.9 Hz, 6H), 0.97 (d, J = 6.6 Hz, 6H).

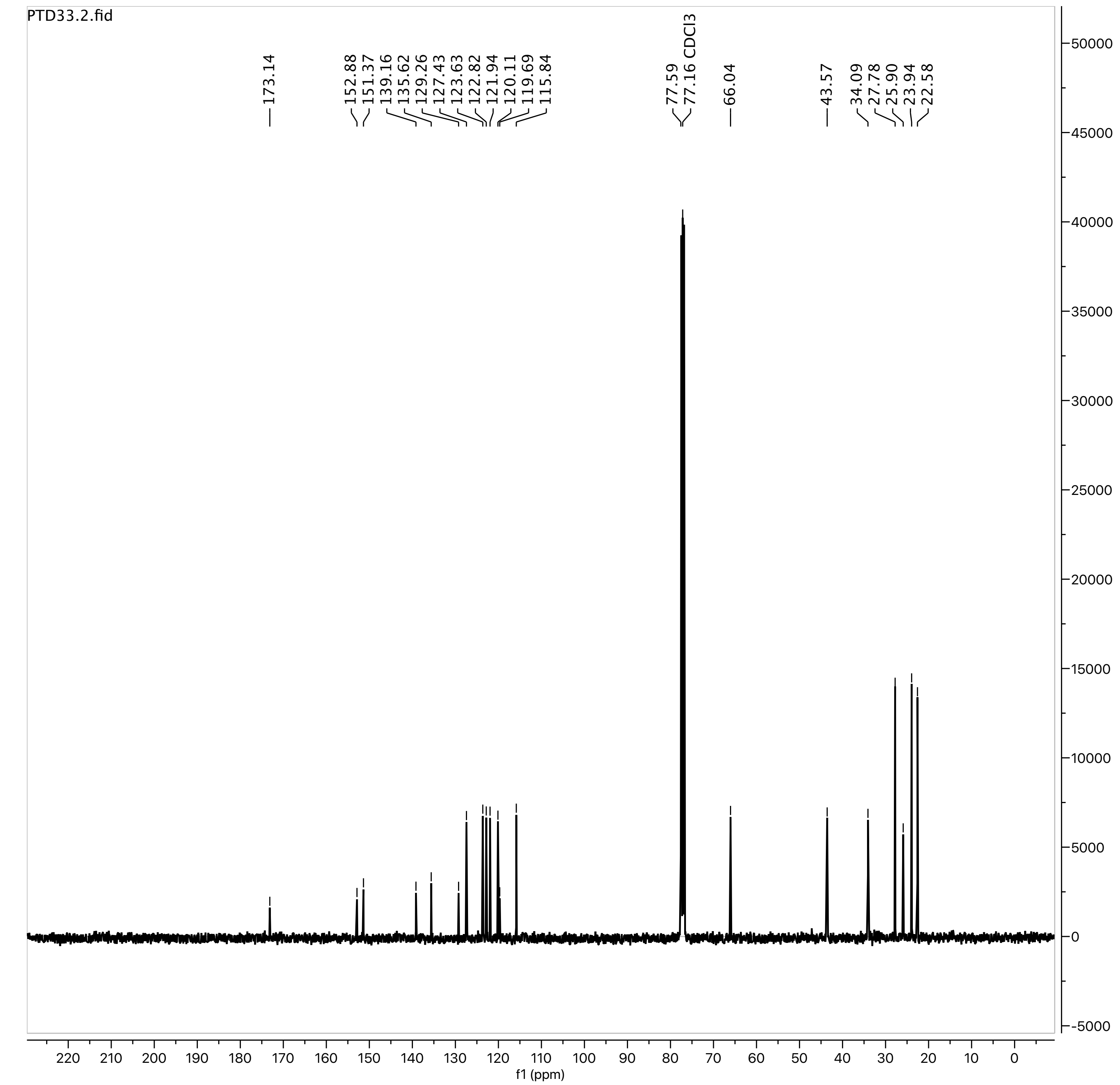

	Parameter	Value
1	Solvent	CDCl3
2	Experiment	1D
3	Spectrometer Frequency	100.62
4	Nucleus	13C

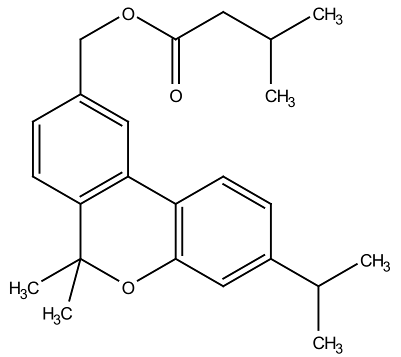

^{13}C NMR (101 MHz, $CDCl_3$) δ 173.14, 152.88, 151.37, 139.16, 135.62, 129.26, 127.43, 123.63, 122.82, 121.94, 120.11, 119.69, 115.84, 77.59, 66.04, 43.57, 34.09, 27.78, 25.90, 23.94, 22.58.

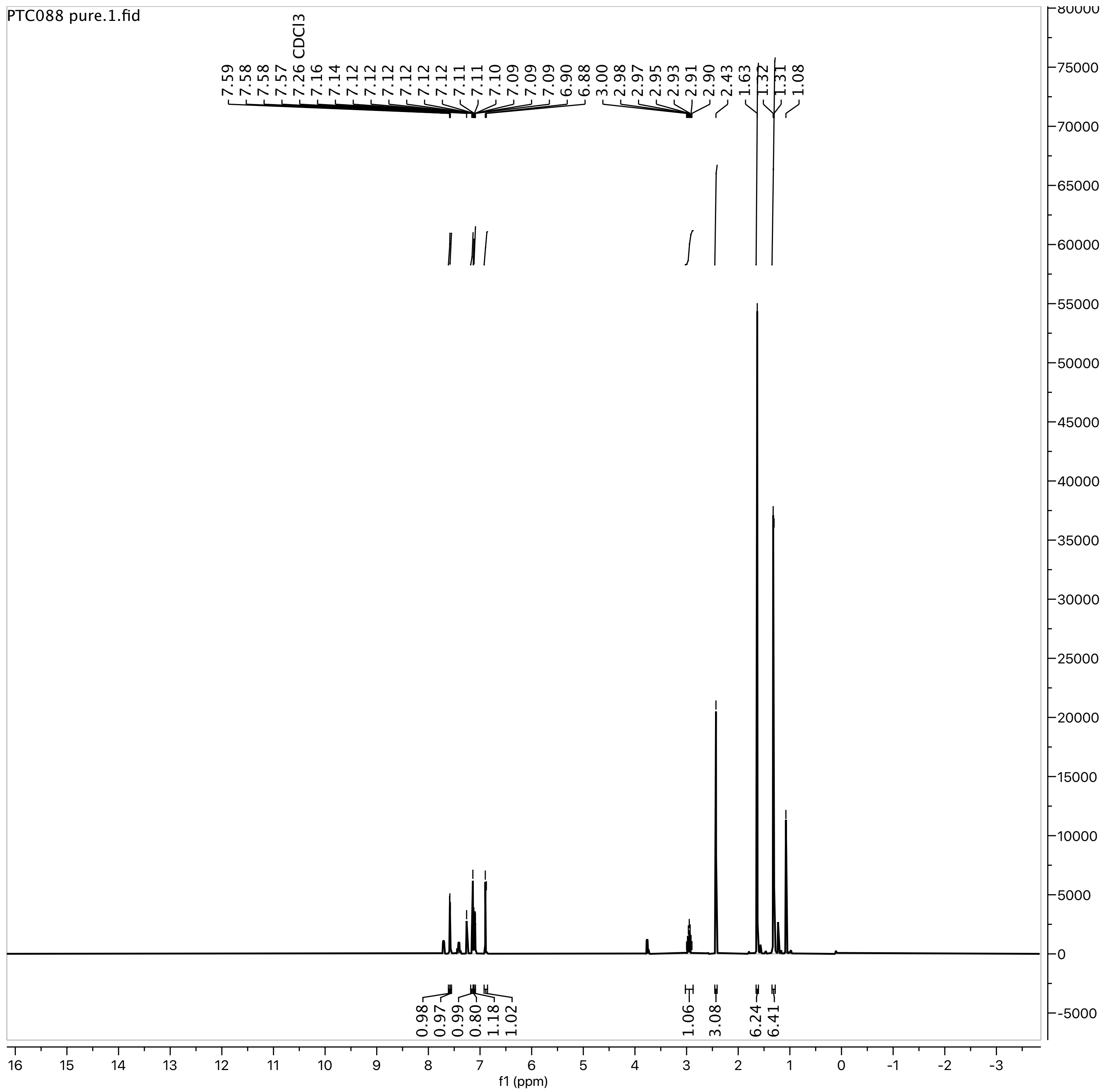

	Parameter	Value
1	Solvent	CDCl3
2	Experiment	1D
3	Spectrometer Frequency	400.13
4	Nucleus	1H

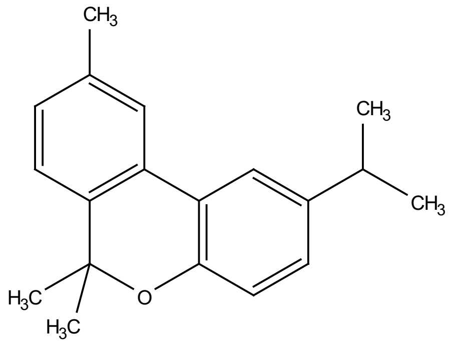

^{1}H NMR (400 MHz, $CDCl_3$) δ 7.59 (d, *J* = 2.2 Hz, 1H), 7.17 – 7.13 (m, 1H), 7.58 (d, *J* = 1.1 Hz, 1H), 7.15 (d, *J* = 7.9 Hz, 1H), 7.12 (dd, *J* = 1.7, 0.7 Hz, 1H), 7.10 (ddd, *J* = 8.2, 2.2, 0.5 Hz, 1H), 6.89 (d, *J* = 8.3 Hz, 1H), 2.95 (hept, *J* = 7.0 Hz, 1H), 2.43 (s, 3H), 1.63 (s, 6H), 1.31 (d, *J* = 6.9 Hz, 6H).

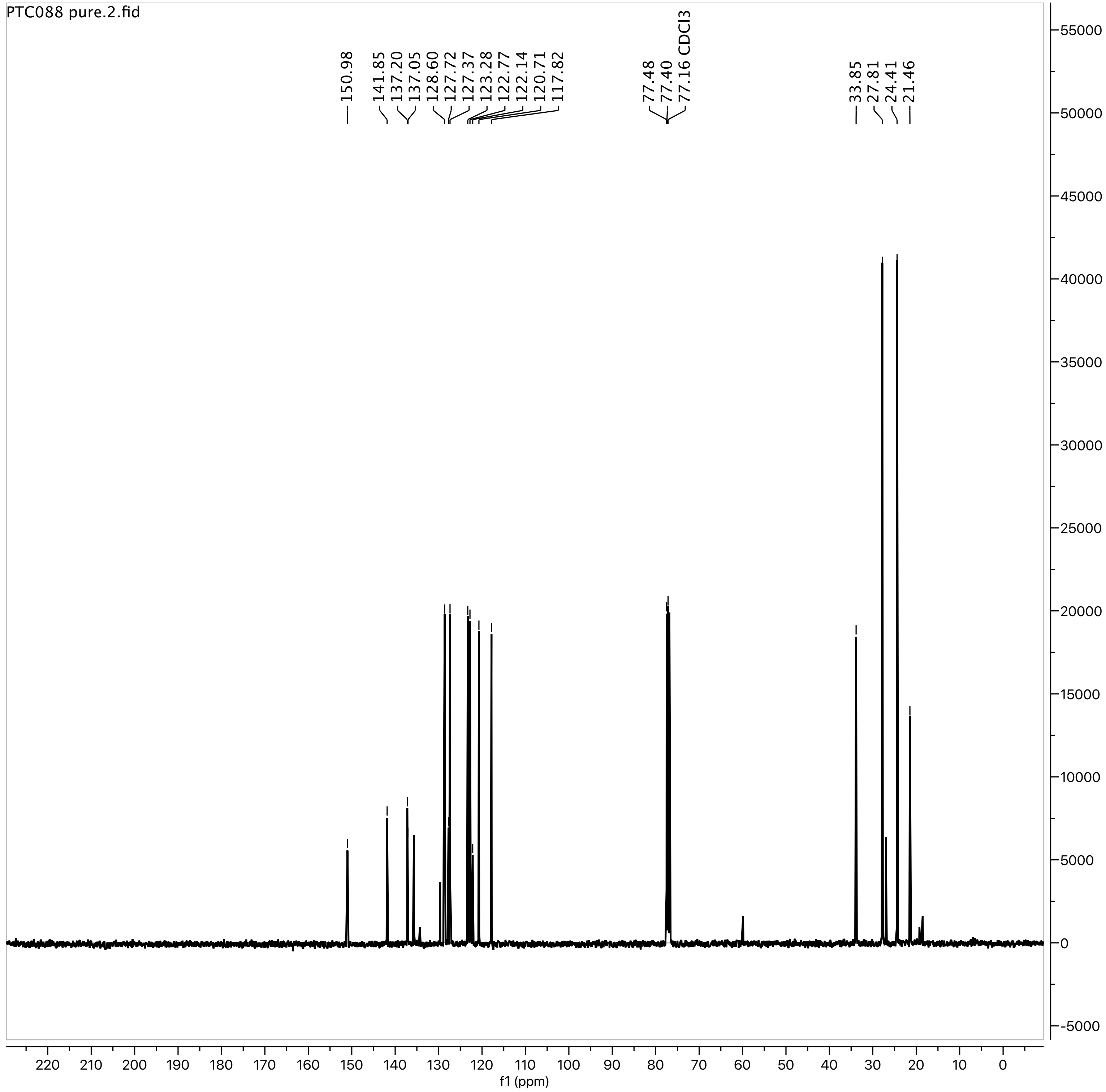

	Parameter	Value
1	Solvent	CDCl3
2	Experiment	1D
3	Spectrometer Frequency	100.62
4	Nucleus	13C

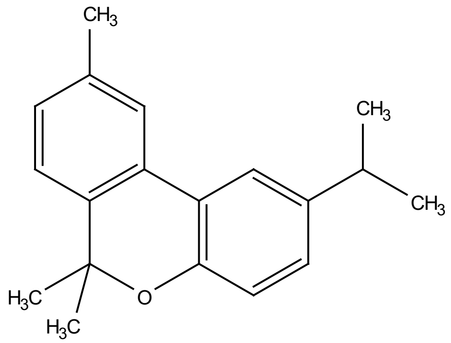

^{13}C NMR (101 MHz, $CDCl_3$) δ 150.98, 141.85, 137.20, 137.05, 128.60, 127.72, 127.37, 123.28, 122.77, 122.14, 120.71, 117.82, 77.40, 33.85, 27.81, 24.41, 21.46.

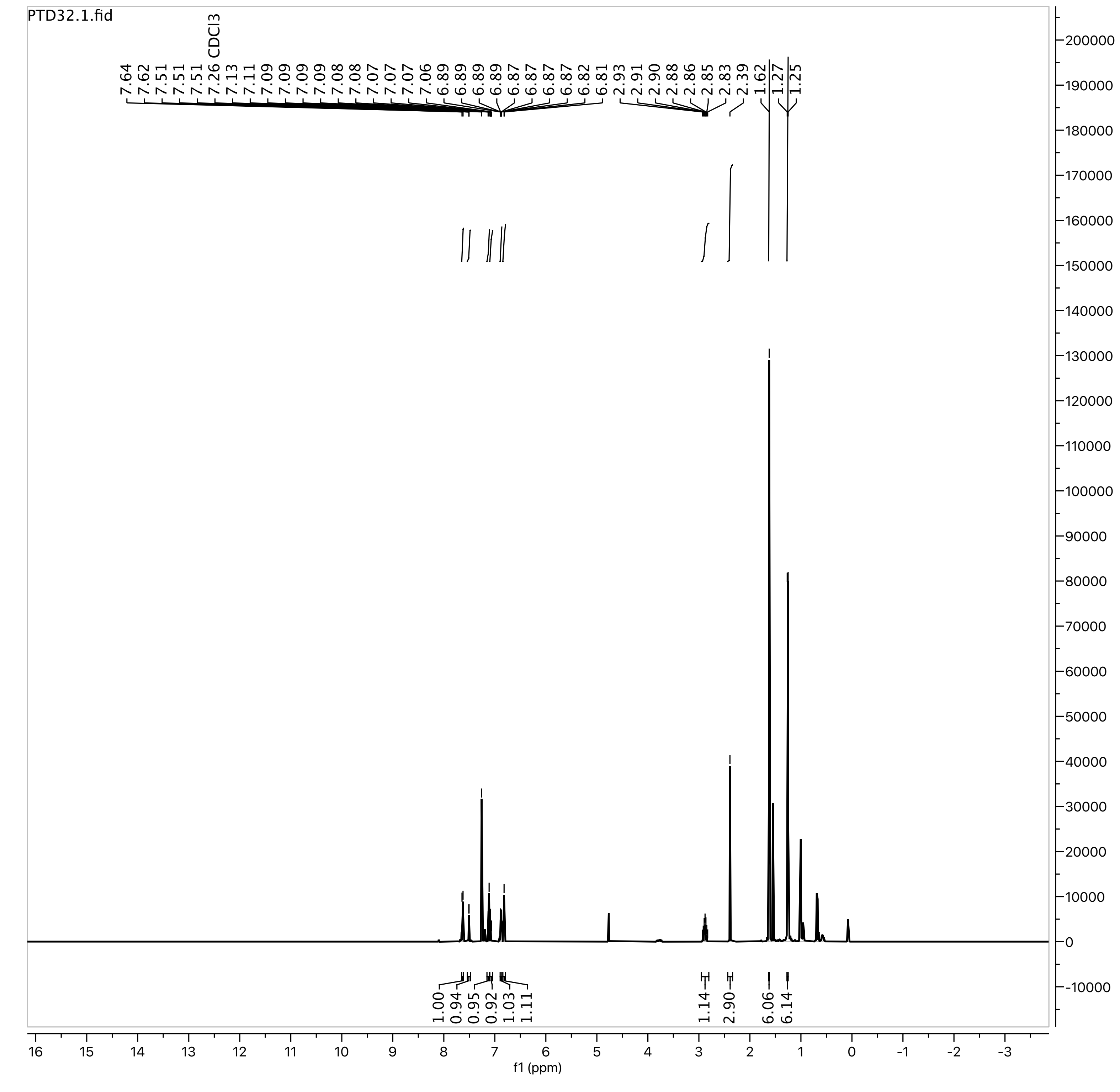

	Parameter	Value
1	Solvent	CDCl3
2	Experiment	1D
3	Spectrometer Frequency	400.13
4	Nucleus	1H

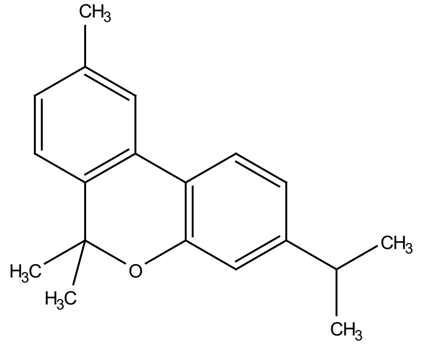

^{1}H NMR (400 MHz, $CDCl_3$) δ 7.63 (d, J = 8.0 Hz, 1H), 7.54 – 7.48 (m, 1H), 7.12 (d, J = 7.8 Hz, 1H), 7.08 (ddd, J = 7.9, 1.7, 0.7 Hz, 1H), 6.88 (ddd, J = 8.0, 1.8, 0.6 Hz, 1H), 6.82 (d, J = 1.8 Hz, 1H), 2.88 (dp, J = 13.8, 6.9 Hz, 1H), 2.39 (s, 3H), 1.62 (s, 6H), 1.26 (d, J = 7.0 Hz, 6H).

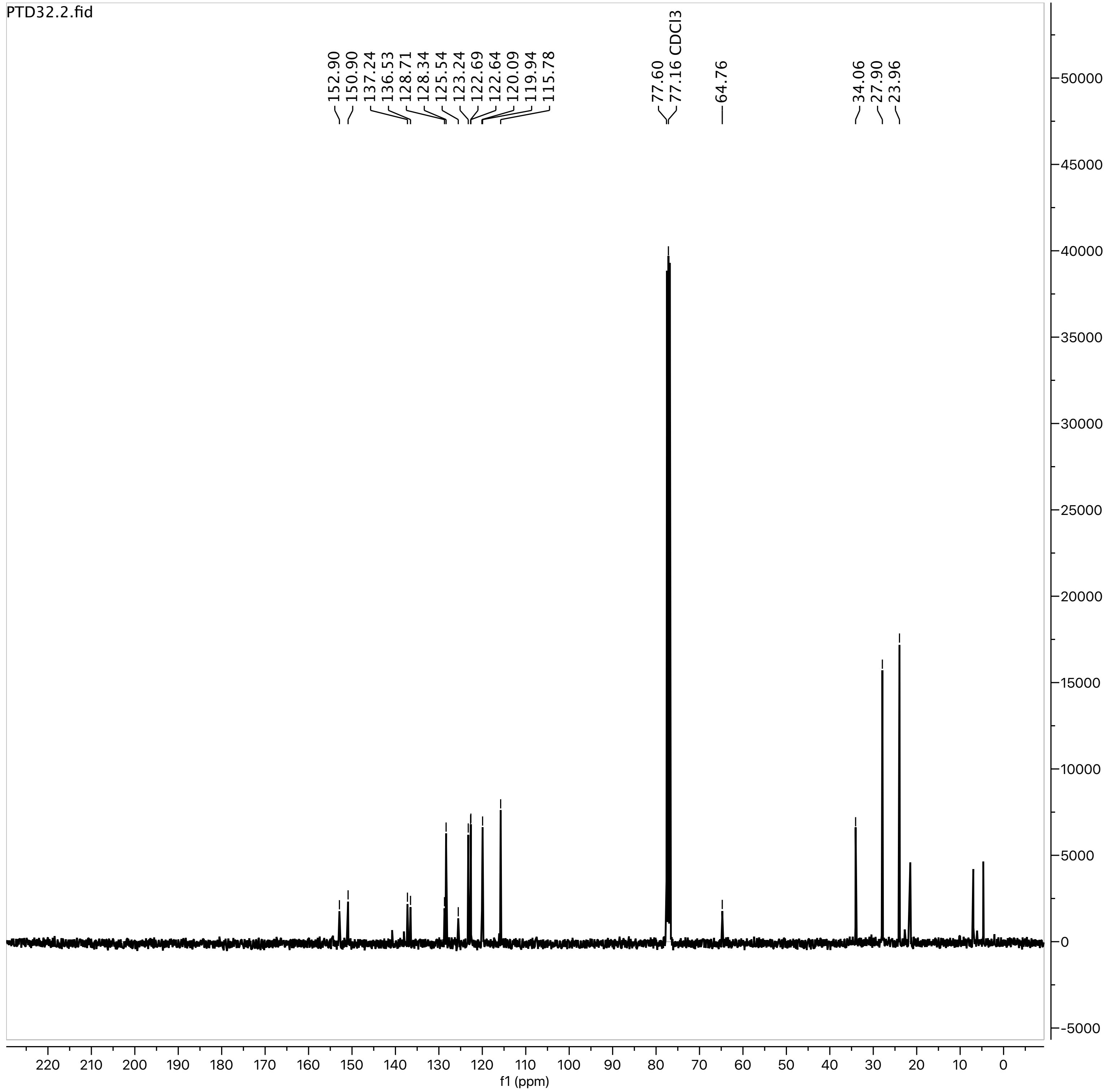

	Parameter	Value
1	Solvent	CDCl3
2	Experiment	1D
3	Spectrometer Frequency	100.62
4	Nucleus	13C

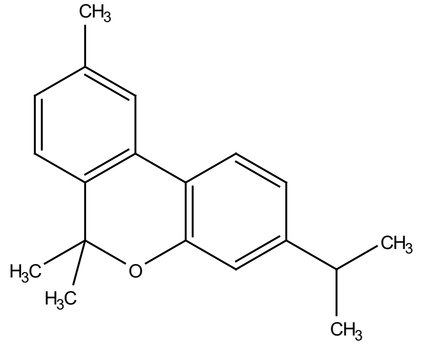

^{13}C NMR (101 MHz, $CDCl_3$) δ 152.90, 150.90, 137.24, 136.53, 128.71, 128.34, 125.54, 123.24, 122.69, 122.64, 120.09, 119.94, 115.78, 77.60, 64.76, 34.06, 27.90, 23.96.

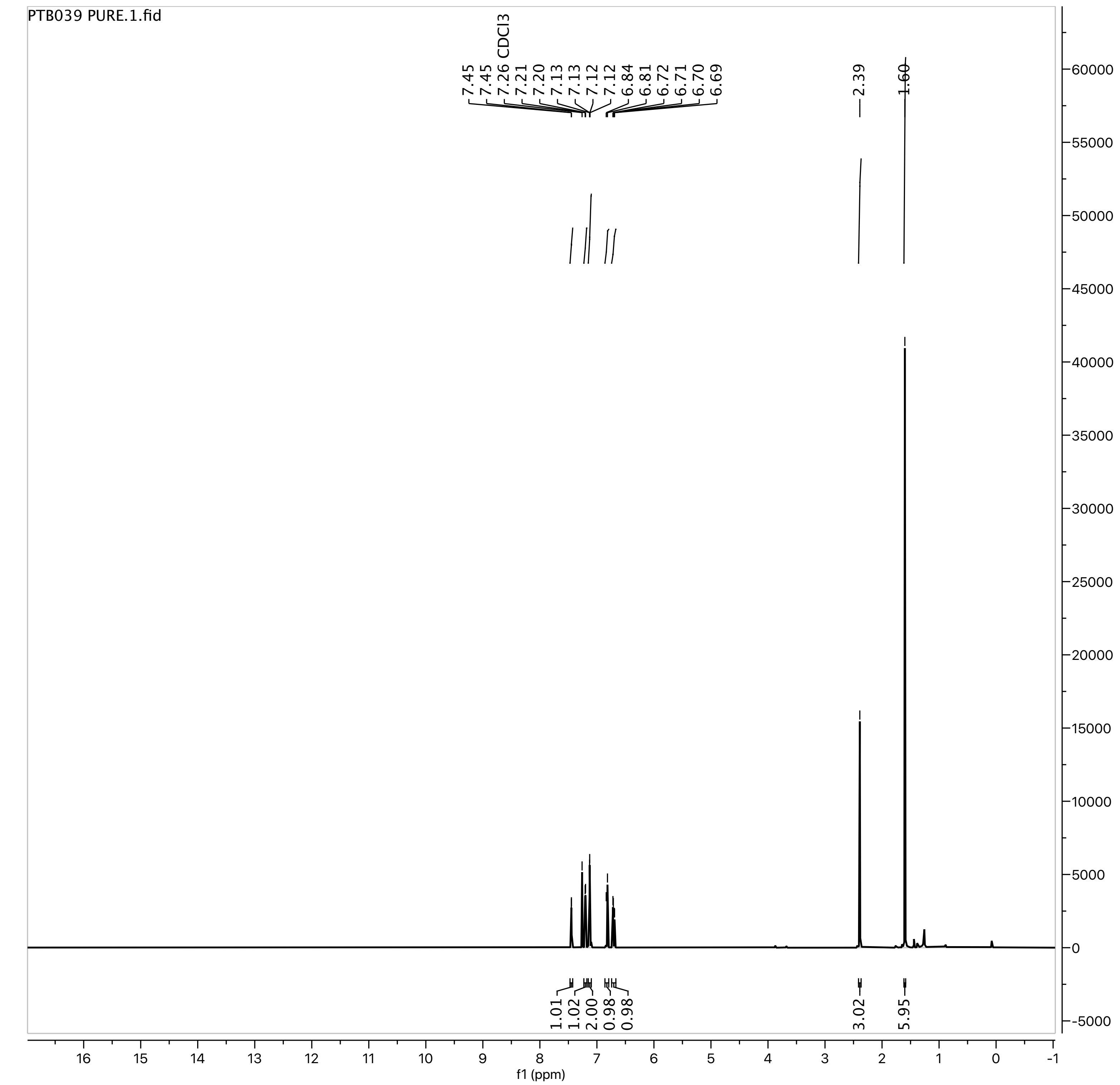

	Parameter	Value
1	Solvent	CDCl3
2	Experiment	1D
3	Spectrometer Frequency	400.13
4	Nucleus	1H

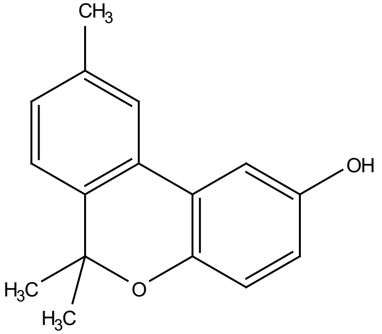

^{1}H NMR (400 MHz, $CDCl_3$) δ 7.47 – 7.42 (m, 1H), 7.20 (d, J = 2.9 Hz, 1H), 7.12 (dd, J = 2.1, 1.0 Hz, 2H), 6.83 (d, J = 8.6 Hz, 1H), 6.70 (dd, J = 8.6, 2.9 Hz, 1H), 2.39 (s, 3H), 1.60 (s, 6H).

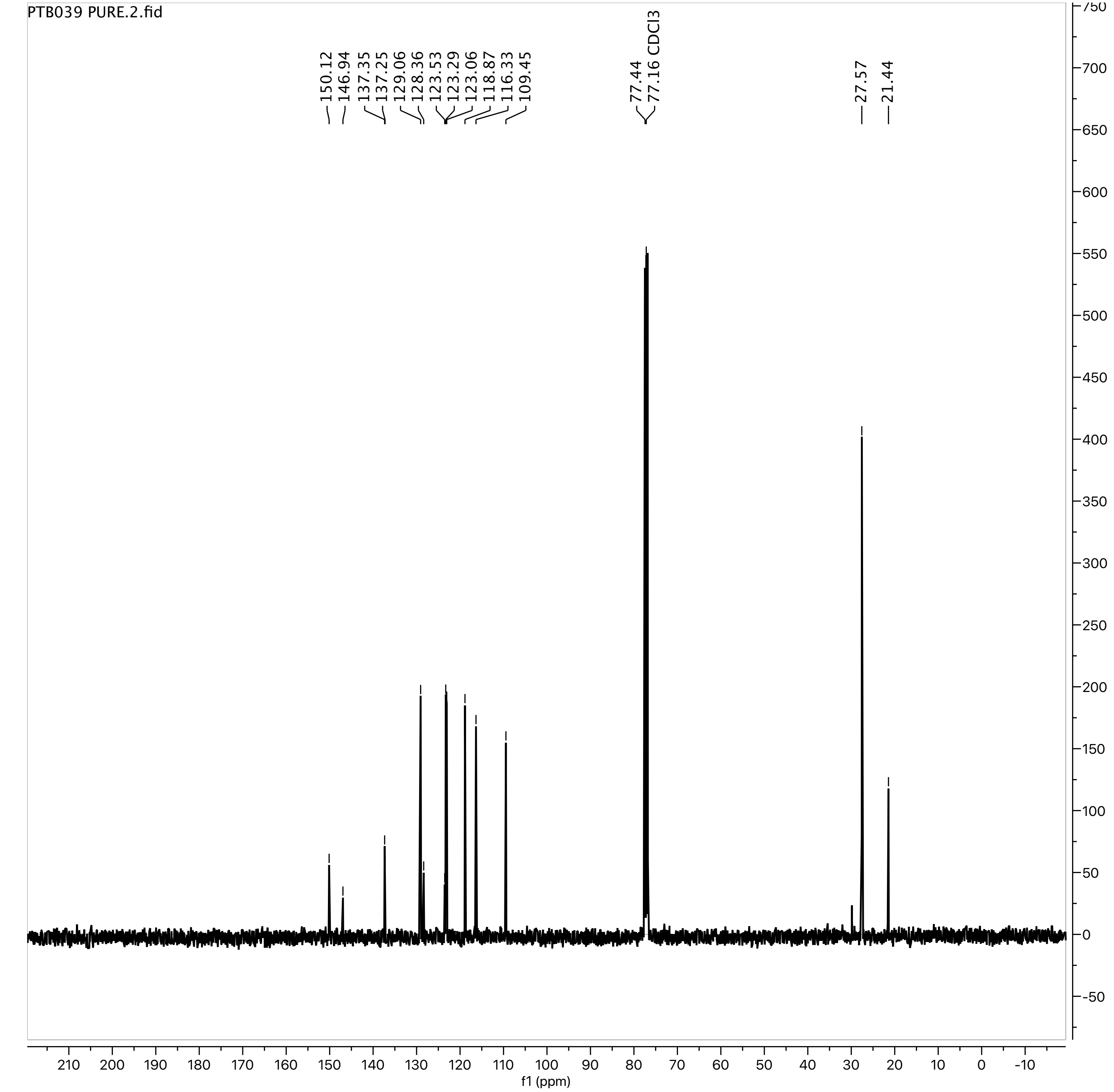

	Parameter	Value
1	Solvent	CDCl3
2	Experiment	1D
3	Spectrometer Frequency	100.62
4	Nucleus	13C

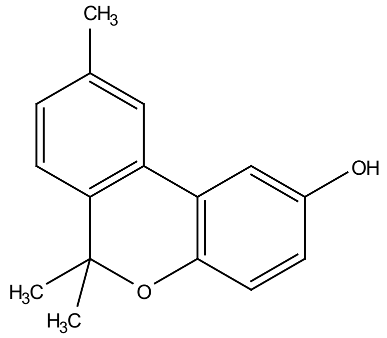

^{13}C NMR (101 MHz, $CDCl_3$) δ 150.12, 146.94, 137.35, 137.25, 129.06, 128.36, 123.53, 123.29, 123.06, 118.87, 116.33, 109.45, 77.44, 27.57, 21.44.

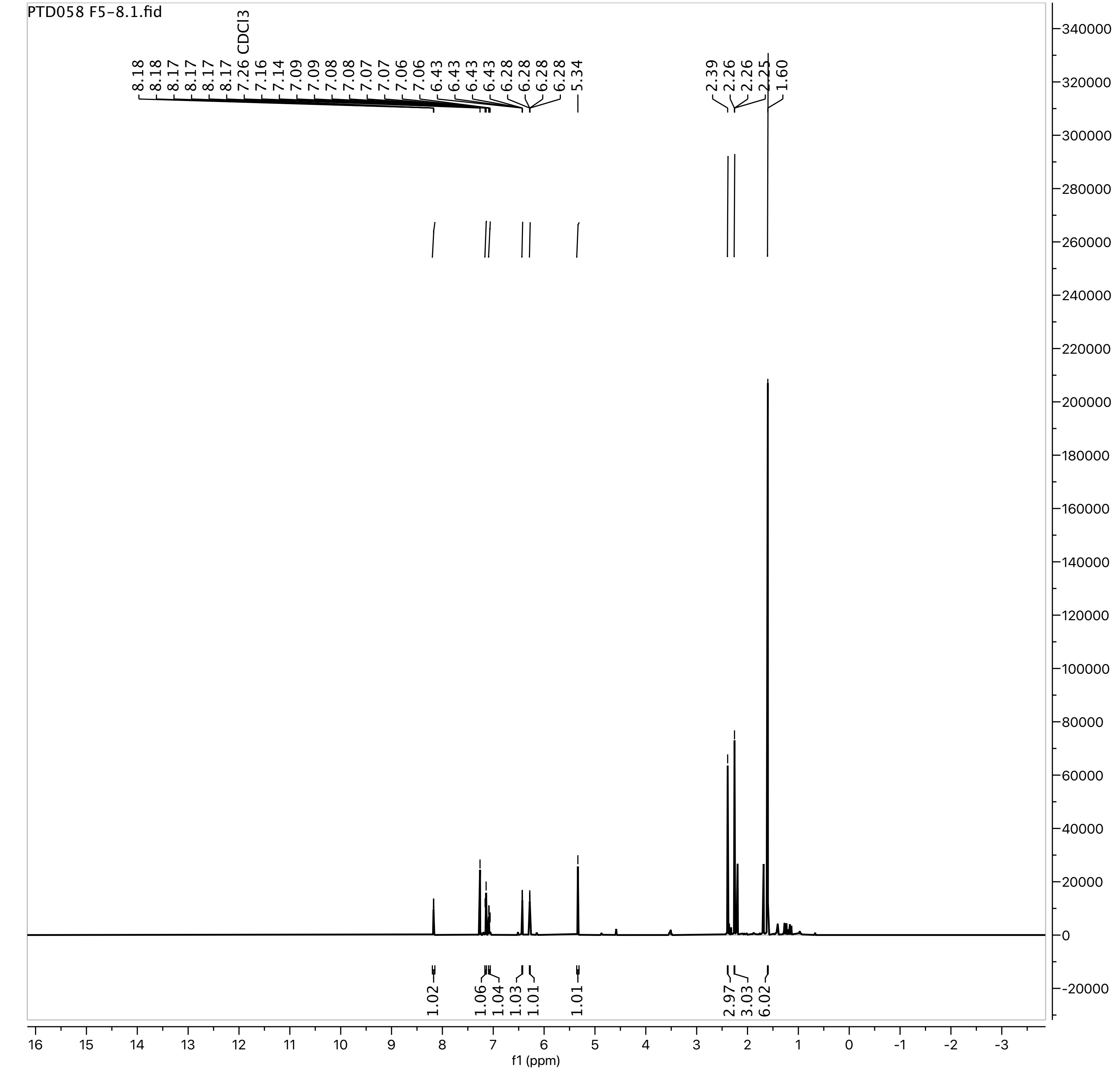

	Parameter	Value
1	Solvent	CDCl3
2	Experiment	1D
3	Spectrometer Frequency	400.13
4	Nucleus	1H

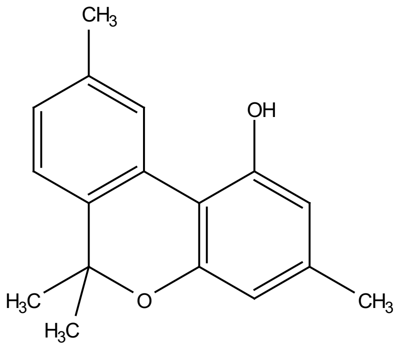

^{1}H NMR (400 MHz, $CDCl_3$) δ 8.17 (dd, *J* = 1.2, 0.6 Hz, 1H), 7.15 (d, *J* = 7.8 Hz, 1H), 7.07 (ddd, *J* = 7.9, 1.8, 0.7 Hz, 1H), 6.43 (dd, *J* = 1.6, 0.7 Hz, 1H), 6.28 (dd, *J* = 1.7, 0.7 Hz, 1H), 5.34 (s, 1H), 2.39 (s, 3H), 2.26 (t, *J* = 0.7 Hz, 3H), 1.60 (s, 6H).

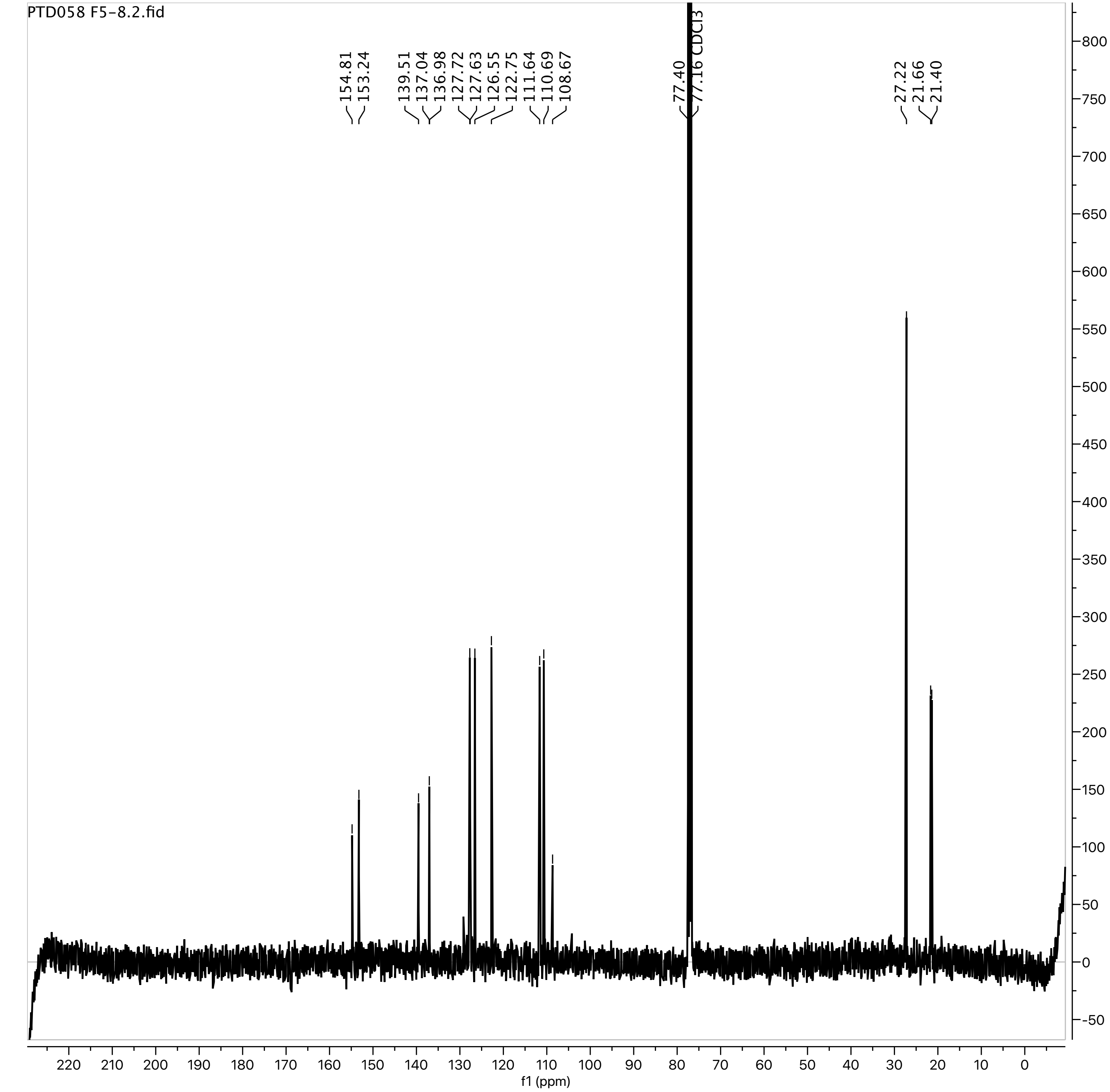

	Parameter	Value
1	Solvent	CDCl3
2	Experiment	1D
3	Spectrometer Frequency	100.62
4	Nucleus	13C

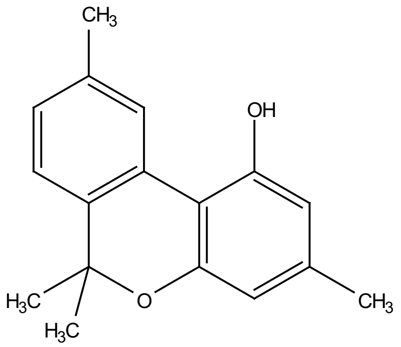

^{13}C NMR (101 MHz, $CDCl_3$) δ 154.81, 153.24, 139.51, 137.04, 136.98, 127.72, 127.63, 126.55, 122.75, 111.64, 110.69, 108.67, 77.40, 27.22, 21.66, 21.40.

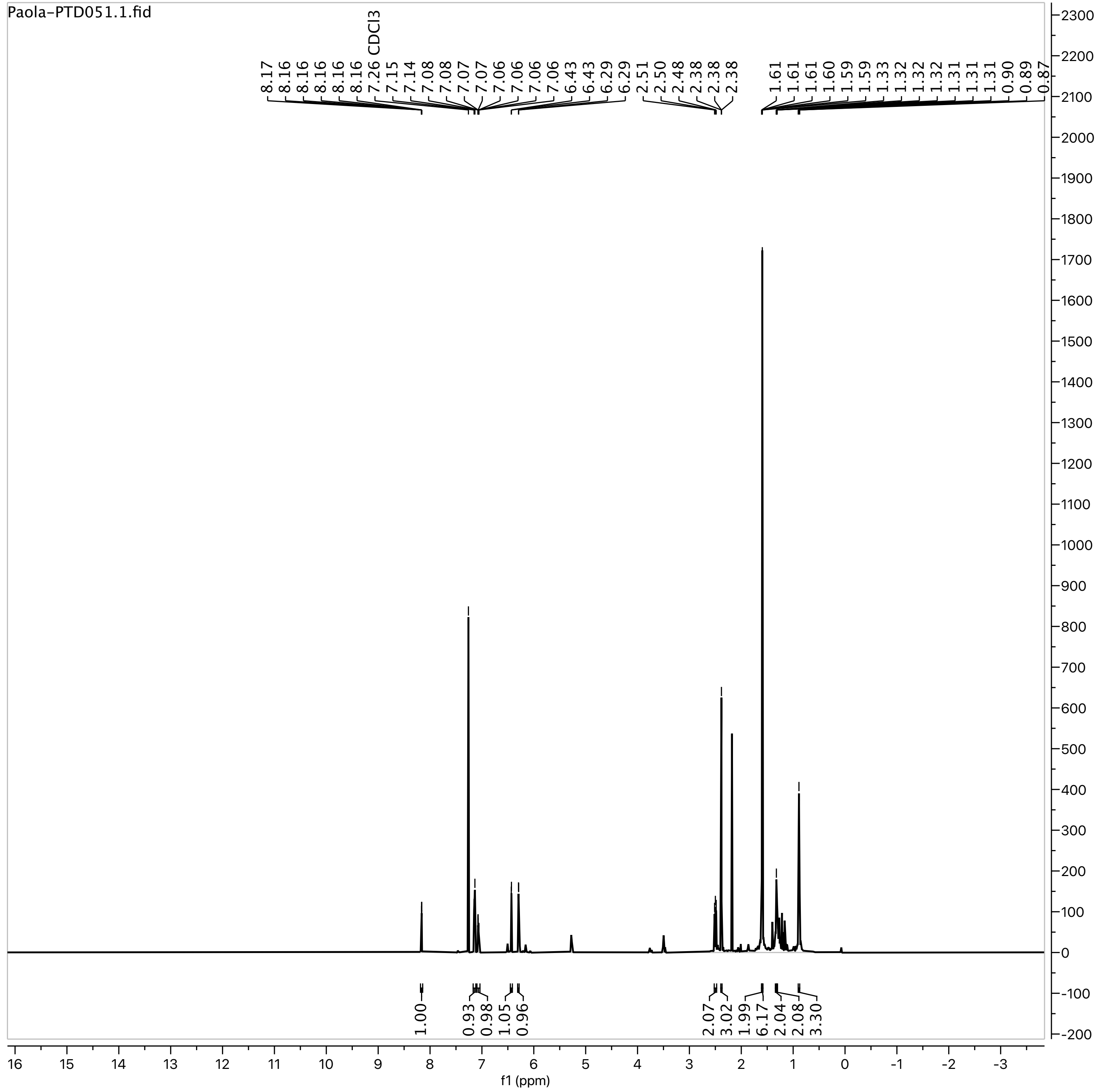

	Parameter	Value
1	Solvent	CDCl3
2	Experiment	1D
3	Spectrometer Frequency	500.17
4	Nucleus	1H

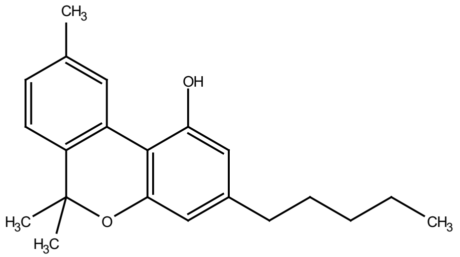

^{1}H NMR (500 MHz, $CDCl_3$) δ 8.18 – 8.14 (m, 1H), 7.14 (d, *J* = 7.8 Hz, 1H), 7.07 (ddd, *J* = 7.8, 1.8, 0.8 Hz, 1H), 6.43 (d, *J* = 1.6 Hz, 1H), 6.29 (d, *J* = 1.6 Hz, 1H), 2.52 – 2.47 (m, 2H), 2.38 (d, *J* = 0.7 Hz, 3H), 1.61 – 1.60 (m, 2H), 1.59 (s, 6H), 1.34 – 1.32 (m, 2H), 1.32 – 1.30 (m, 2H), 0.90 – 0.87 (m, 3H).

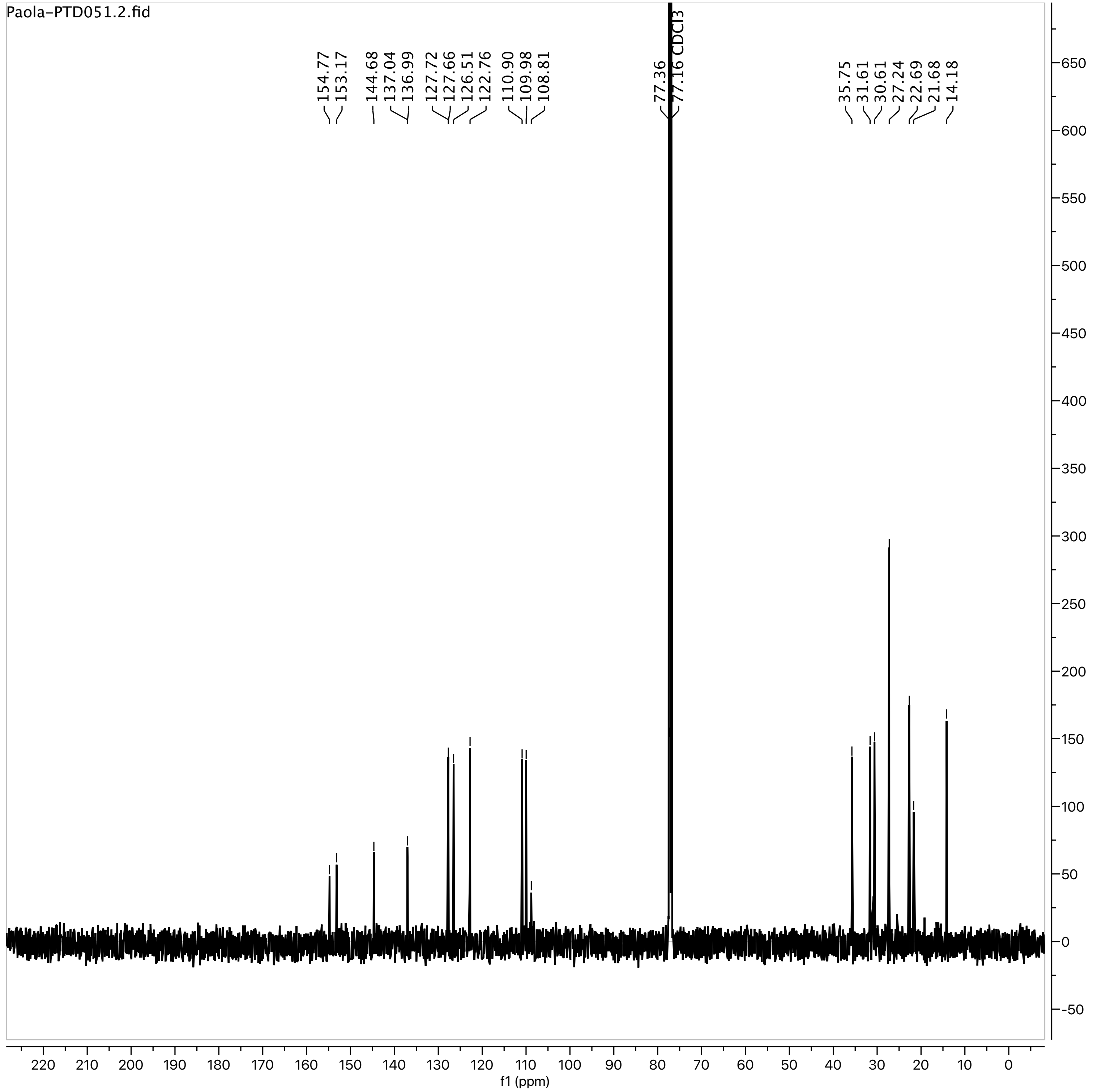

	Parameter	Value
1	Solvent	CDCl3
2	Experiment	1D
3	Spectrometer Frequency	125.78
4	Nucleus	13C

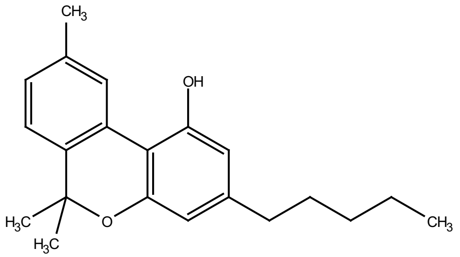

^{13}C NMR (126 MHz, $CDCl_3$) δ 154.77, 153.17, 144.68, 137.04, 136.99, 127.72, 127.66, 126.51, 122.76, 110.90, 109.98, 108.81, 77.36, 35.75, 31.61, 30.61, 27.24, 22.69, 21.68, 14.18.
